# Hybrid Extracellular Vesicles for Efficient Loading and Functional Delivery of mRNA

**DOI:** 10.1002/jev2.70201

**Published:** 2025-12-14

**Authors:** Xiaoqin Wang, Michael J. Munson, Kristina Friis, Anna Marzeda, Andreia M. Silva, Franziska Kohl, Leif Hultin, Raymond M. Schiffelers, Niek Dekker

**Affiliations:** ^1^ Discovery Biology, Discovery Sciences BioPharmaceuticals R&D, AstraZeneca Gothenburg Sweden; ^2^ Advanced Drug Delivery, Pharmaceutical Sciences BioPharmaceuticals R&D, AstraZeneca Gothenburg Sweden; ^3^ Discovery Bioanalysis, Clinical Pharmacology and Safety Sciences BioPharmaceuticals R&D, AstraZeneca Gothenburg Sweden; ^4^ Genomic Research, Discovery Sciences BioPharmaceuticals R&D, AstraZeneca Gothenburg Sweden; ^5^ Imaging Sciences, Clinical Pharmacology and Safety Sciences BioPharmaceuticals R&D, AstraZeneca Gothenburg Sweden; ^6^ CDL Research University Medical Center Utrecht Utrecht the Netherlands

**Keywords:** cell tolerability, endosomal escape, functional biodistribution, functional delivery, hybrid extracellular vesicle, mRNA loading

## Abstract

Extracellular vesicles (EVs) are an attractive delivery vehicle with biological activity, intrinsic homing, low immunogenicity, and engineerability; however, challenges remain regarding loading and functional delivery of mRNA. Here, we developed a novel approach to load mRNA through low pH‐induced fusion of EVs with lipid nanoparticles (LNPs) to generate hybrid EVs (HEVs). Conventional characterization showed that HEVs preserved classical features of EVs. Single particle analysis revealed successful loading of mRNA and incorporation of LNP components into HEVs. The combined properties from EV and LNP contributed to the excellent cell tolerability of HEV, overcoming dose‐limit toxicity, and functional delivery of mRNA by HEV. We further elucidated the mechanism of HEV‐mediated intracellular delivery of mRNA. Our results showed that in contrast to source EVs, HEVs were capable of inducing endosomal escape, facilitating intracellular delivery of mRNA. Furthermore, HEVs functionally delivered mRNA *in* *vivo* and displayed extrahepatic delivery capacity with predominant functional distribution in spleen. Our results suggest HEVs as a promising EV‐based delivery platform for mRNA delivery.

## Graphical abstract

Hybrid extracellular vesicles (HEV) produced by EV‐LNP fusion enable efficient loading and functional delivery of mRNA. HEV integrated properties of both EV and LNP contributing to their excellent cell tolerability and acquired capacity of inducing endosomal escape. HEV demonstrated functional delivery of mRNA in mice and displayed extrahepatic delivery capacity with predominant functional distribution in spleen.

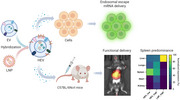

## 1 Introduction

Extracellular vesicles (EVs) are protein‐rich membrane enclosed nanovesicles naturally secreted by almost all cell types and present in various biofluids (Yáñez‐Mó et al. 2015). EVs carry a broad range of bioactive cargoes and more importantly these cargoes can be functionally transferred among cells to mediate cell‐to‐cell communication (Kalluri and LeBleu 2020). Accumulating evidence reveals that EVs display various functional features such as immunomodulatory effects (Gomzikova et al. 2019; Massaro et al. 2021) and regenerative properties (Bollini et al. 2018; Wang and Thomsen 2021). The concept to utilize EVs as a therapeutic is based on the findings of EVs’ intrinsic functions. Several popular EVs, for example EVs from mesenchymal stem cells (Wang et al. 2018, 2020), immune cells (Dosil et al. 2022; Ekström et al. 2013) and cancer cells (Hoshino et al. 2015; Zhou et al. 2022), are intensively studied and their therapeutic application is being explored accordingly. While the therapeutic potential of EVs is demonstrated, it is not yet completely clear the mechanism of action of naïve EV‐mediated functions and the quantity of therapeutic relevant contents in naïve EVs (Gupta et al. 2021; Toh et al. 2018). Moreover, challenges remain on production of sufficient amounts of EVs to fulfill the doses estimated to result in therapeutic effects, especially for clinical application (Gupta et al. 2021) and hence hinder the clinical translation of EV therapeutics. These together highlight the need of enhancing EVs’ therapeutic effects by equipping them with exogenously loaded cargoes. From the other perspective, the innate features of EVs and feasibility of engineering EVs raise a promising opportunity to develop EVs as the next generation of delivery vehicle. EV‐based delivery vehicles may potentially overcome some of the current limitations of synthetic delivery vehicles, such as immunogenicity (Faksova et al. 2024). Owing to their natural origin, EVs from cell sources such as Expi293F show low immunogenicity and toxicity (Saleh et al. 2019). Moreover, recent evidence suggest that EVs are capable of crossing biological barriers such as the blood‐brain barrier (Krämer‐Albers 2022) though challenges remains on limited delivery efficiency in brain especially when EVs were administrated intravenously (reviewed by Williams et al. (2025)). EVs were reported to penetrate and accumulate in preferential organs such as liver and lungs, which is possibly due to their propagation of the surface properties of cells (Hoshino et al. 2015). These observations shed light on the hope that EV‐based delivery vehicles may be able to increase cellular uptake and targeting specificity of delivery and thus enhance delivery efficiency. Nevertheless, successful loading and functional transfer of the exogenous cargoes of interest is critical for the future success of EV‐based delivery and development in the context of therapeutic applications.

Currently, there are two main strategies for cargo loading into EVs: endogenous and exogenous loading. Endogenous loading mainly relies upon genetic modification of the cells of origin to overexpress the nucleic acid or protein cargo of interest in an attempt to transfer them from the parental cells to secreted EVs. However, major challenges remain for this strategy on efficient packaging of the cargo of interest into EVs and limitations on the size of the desired cargo. Although recent advances utilizing the specific interaction of RNA binding proteins, such as hnRNPA2B1, and EXOmotifs or zipcode sequences to achieve active loading of RNA in EVs, these approaches favor the loading of small RNA than large RNA molecules (Hung and Leonard 2016; Kanada et al. 2015; Ramanathan et al. 2019) and the loading efficiency was low or rarely reported (Kanada et al. 2015; Sutaria et al. 2017). Similar challenges exist in some of exogenous loading approaches, such as electroporation (Usman et al. 2018; Kooijmans et al. 2013; Lamichhane et al. 2015) and sonication (Lamichhane et al. 2016), which often limit to loading of small cargos, including siRNA, miRNA and small molecule drugs, and various loading efficiencies are reported (Rankin‐Turner et al. 2021). Additionally, major concerns on preserving the quality of EVs and the exogenous cargo using these approaches and quantification of the truly encapsulated exogenous cargo were raised. For example, electroporation was shown to result in aggregation of EVs (Hood et al. 2014; Johnsen et al. 2016) as well as siRNA, which negatively impacts the integrity of EVs, therapeutic function of siRNA, and may lead to inaccurate measurement of loading efficiency (Kooijmans et al. 2013). Given the limitations addressed, achieving successful and efficient loading of large cargoes such as mRNA in EVs while preserving the biological identity of EVs and quality of loaded cargoes remains challenging.

Previously, EVs have been shown to functionally deliver cargo (O'Brien et al. 2022; Ratajczak et al. 2006), but in an inefficient manner (Hung and Leonard 2016; Kanada et al. 2015; O'Brien et al. 2022). This may be due to the complexity of how EVs interact with cells and release their cargo intracellularly. EVs can enter the cells via multiple routes, among which clathrin/caveolin‐mediated endocytosis is suggested to be the main route (Mulcahy et al. 2014). Therefore, the fates of EVs in the endosomal path is critical to determine if their cargoes can be functionally transferred to the cytosol. Degradation, recycling or re‐release of EVs and EV cargo may be reasons leading to non‐functional delivery observed previously (Hung and Leonard 2016; Kanada et al. 2015). Recent studies revealed a novel fate that EVs were retained in the endosomal compartment and eventually degraded or re‐released (O'Brien et al. 2022; Mathieu et al. 2019). These findings indicate the capacity of EVs to escape from endosomes is key for efficient functional delivery and therapeutic effects mediated by EVs.

Highlighting these challenges shed light on the need to develop an EV‐based delivery platform that allows loading and functional delivery of large cargo, such as mRNA. To achieve such a goal, in this study we implement the emerging concept to integrate the advantages of natural and synthetic nanoparticles, in particular EVs and lipid nanoparticles (LNPs), to produce hybrid EVs (HEVs) for loading of mRNA. We demonstrated successful loading of mRNA in HEV at a single particle level and high encapsulation efficiency of the loaded mRNA. We comprehensively characterized the features of HEVs at both bulk population and single particle resolution by complementary techniques. The cell tolerability and functional delivery of mRNA by HEVs were evaluated across multiple cell lines. The mechanism of HEV‐mediated intracellular delivery of mRNA was further illustrated by investigation of endosomal escape. Moreover, we validated the functional delivery of mRNA by HEVs *in* *vivo* and examined the functional biodistribution of HEV‐delivered mRNA. Our results revealed that HEVs are a promising approach for efficient loading and functional delivery of mRNA.

## 2 Materials and Methods

### 2.1 Cell Culture

Expi293F cells (ThermoFisher Scientific, A14527) derived from the human embryonic kidney 293 cell line were seeded in chemically defined, serum‐ and protein‐free Expi293 expression media (ThermoFisher Scientific) at a density of 0.5–1.0 × 10^6^ cells/mL. The culture was maintained in an incubator with a setting of 37°C, 8% CO_2_ and shaking speed at 125 rpm. Expi293F cell density and viability were monitored by Cedex HiRes Analyzer (Roche Diagnostics). When cells reached a density of ∼4.0 × 10^6^ cells/mL, Expi293F conditioned media (CM) was collected by centrifugation of cell suspension at 300 × *g* for 10 min and at 2500 × *g* for 30 min, 4°C to remove cells and cell debris, respectively. The clarified CM (CCM) was stored at –80°C until use for EV isolation.

### 2.2 EV Isolation

EVs were isolated from Expi293F CCM by differential ultracentrifugation using a 45Ti Rotor and an Optima XE‐100 ultracentrifuge (Beckman Coulter). Briefly, Expi293F CCM was transferred to polyallomer 94 mL Quick‐Seal ultracentrifuge tubes (Beckman Coulter) and underwent centrifugation at 20,000 × *g* for 25 min at 4°C to eliminate large EVs. The supernatant was carefully transferred to new tubes and centrifuged at 100,000 × *g* for 120 min at 4°C to pellet small EVs. The small EV pellet was resuspended in sterile PBS and stored at –80°C until use.

### 2.3 LNP Formulation

DLin‐MC3‐DMA LNPs were formulated using a NanoAssemblr Ignite (Precision NanoSystems) with a microfluidic mixing chip. Briefly, the lipid mixture was prepared in 99% ethanol at the molar ratio of 50:38.5:10:1.5 (MC3: Cholesterol: DSPC:DMPE‐PEG2000). Cre mRNA (TriLink, L7211), unlabeled and Cy5‐labelled EGFP mRNA (TriLink, L7201/7701, 4:1 ratio) were prepared in RNase free 50 mM citrate pH 3 buffer (TekNova, Q2445). Lipids and mRNA cargo were prepared at a ratio of ∼10:1 (w/w), N:P ratio of 3:1. To form nanoparticles, the two solutions containing lipids and mRNA respectively were mixed 3:1 at a constant flow rate of 12 mL/min. Subsequently, particles were dialyzed overnight using a Slider‐A‐Lyzer G2 dialysis 10K molecular weight cut‐off cassette (Thermo Scientific) in PBS (Thermo Fisher) pH 7.4, at 4°C and sterilized by filtration using a 0.2 µm filter.

### 2.4 Method Development of HEV Production

HEVs were produced by mixing of EVs and LNPs at specific particle ratios and concentrations. Particle fusion was induced by lowering the pH of the particle mixture using MES buffer pH 5.5 and incubated at room temperature (RT) for 30 min. Fusion reaction with only LNPs served as control. Post incubation the pH of the particle mixture was raised up using PBS pH 7.4 and HEVs were recovered by ultracentrifugation at 100,000 × *g* for 120 min at 4°C. To optimize this procedure, different concentrations of particle mixture, ranging from 10^10^ to 10^12^ particles per millilitre, and EV:LNP particle ratios, ranging from 3:1 to 1:3, were evaluated. Quality of HEVs produced at different conditions, in terms of the recovery efficiency, size profile and RNA encapsulation efficiency, were compared for selection of the optimal production condition. HEV samples were freshly prepared prior to each experiment.

### 2.5 Quantification of Particles and Profile of Size Distribution

The particle concentration and size distribution of EVs, LNPs and HEVs were analyzed by Nanoparticle tracking analysis (NTA) using NanoSight LM14c (Malvern Panalytical) equipped with a CMOS camera (Hamamatsu Photonics). Samples were prepared in PBS at the desired dilution and injected into the measuring chamber at a constant speed controlled by a syringe pump. Particles were tracked under flow, and each sample was recorded for three videos of 90 s duration each. Equipment settings were kept constant among measurements with camera level set at 16 and screen gain set at 1. Data analysis was performed using NTA 3.2 software (Malvern Panalytical) with detection threshold set to 5. The particle recovery efficiency of HEV was calculated as the following:

$$\begin{array}{ccc} & & \mathrm{Particle}\;\mathrm{recovery}\;\mathrm{efficiency}\;\left(\%\right)\\ & & \;=\frac{\mathrm{Particle}\;\mathrm{number}\;\mathrm{of}\;\mathrm{HEV}}{\mathrm{Particle}\;\mathrm{number}\;\mathrm{of}\;\mathrm{input}\;\mathrm{EV}\;\mathrm{in}\;\mathrm{fusion}\;\mathrm{reaction}}\times 100 \end{array}$$

### 2.6 Quantification of RNA and Analysis of RNA Encapsulation

RNA loaded in LNP and HEV was quantified by Qubit RNA HS quantification assay (ThermoFisher Scientific) according to the manufacturer's instructions. RNA quantification was performed with or without lysis of the particles in 1% Triton X‐100. RNA amount obtained with particle lysis was referred to as total RNA. The RNA recovery efficiency of HEV and RNA encapsulation efficiency were calculated as follows:

$$\begin{array}{ccc} & & \mathrm{RNA}\;\mathrm{recovery}\;\mathrm{efficiency}\;\left(\%\right)\\ & & \;=\frac{\mathrm{Total}\;\mathrm{RNA}\;\mathrm{loaded}\;\mathrm{in}\;\mathrm{HEV}}{\mathrm{Total}\;\mathrm{RNA}\;\mathrm{of}\;\mathrm{input}\;\mathrm{LNP}\;\mathrm{in}\;\mathrm{fusion}\;\mathrm{reaction}}\times 100 \end{array}$$

$$\begin{array}{ccc} & & \mathrm{RNA}\;\mathrm{encapsulation}\;\mathrm{efficiency}\left(\%\right)\\ & & \;=\left(1-\frac{\mathrm{RNA}\;\mathrm{amount}\;\mathrm{without}\;\mathrm{particle}\;\mathrm{lysis}}{\mathrm{RNA}\;\mathrm{amount}\;\mathrm{with}\;\mathrm{particle}\;\mathrm{lysis}}\right)\times 100 \end{array}$$

### 2.7 Immunoelectron Microscopy

Six microliters of EV, HEV or LNP suspension were fixed in 2% paraformaldehyde (Sigma‐Aldrich) for 30 min. Glow‐discharged carbon‐coated nickel grids were placed on the top of the sample drops and incubated at RT for 15 min. The grids with adhered particle samples were washed with 0.1 M PBS and blocked in buffer with 0.1 M glycine and 0.3% BSA for 10 min. After blocking, the grids were incubated at RT for 1 h with primary antibody mixture containing mouse anti‐CD63 (Abcam, ab59479, 1:100) and rabbit anti‐PEG (Abcam, ab190652, 1:500). Following a second blocking step for 20 min, the grids were incubated for 1 h with a mixture of goat anti‐mouse IgG (Abcam, ab39614), 1:20) and goat anti‐rabbit IgG (Abcam, ab27236, 1:20) secondary antibodies conjugated with 6 and 15 nm gold particles, respectively. Finally, the grids were washed and negatively stained with 2% aqueous uranyl acetate for observation under the transmission electron microscope FEI Tecnai G2 Spirit (ThermoFisher Scientific). Images were taken with a Xarosa digital camera (EMSIS GmbH) controlled by Radius v.2.1 software.

### 2.8 Western Blotting Analysis

Protein concentration of EV and HEV samples were quantified using Qubit protein assay kit (ThermoFisher Scientific) according to the manufacturer's instruction. The same amount of total protein from each sample was mixed with NuPAGE LDS sample buffer with or without reducing reagent (ThermoFisher Scientific) and denatured at 75°C for 10 min followed by separation on NuPAGE 4%–12% Bis‐tris gels (ThermoFisher Scientific) and blotting on PVDF membranes (Bio‐Rad Laboratories) using a Trans‐Blot Turbo Transfer System (Bio‐Rad Laboratories). Membranes were then blocked with Odyssey tris‐buffered saline (TBS) buffer (LI‐COR) for 1 h at RT before incubation with primary antibodies overnight at 4°C. The following primary antibodies were diluted in Odyssey TBS buffer for use: mouse anti‐CD63 (Abcam, ab59479, 1:1000); mouse anti‐CD81 (Abcam, ab79559, 1:1000); rabbit anti‐TSG101 (Abcam, ab30871, 1:1000); mouse anti‐Alix (Abcam, ab117600, 1:1000); rabbit anti‐calnexin (Abcam, ab22595, 1:1000) and mouse anti‐β‐actin (Sigma‐Aldrich, A1978, 1:5000). Subsequently, membranes were washed three times with TBS‐Tween20 0.05% (TBS‐T, Sigma‐Aldrich) and incubated with anti‐mouse or anti‐rabbit fluorophore‐conjugated secondary antibodies (LI‐COR, 1:20,000) diluted in TBS‐T for 45 min at RT. After incubation membranes were washed three times with TBS‐T and visualized with an Odyssey CLx imaging system (LI‐COR). Images were processed in the Image Studio v.4.0 software (LI‐COR). Three batches of samples were analyzed respectively in three independent experiments.

### 2.9 Nanoflow Cytometry Analysis

HEVs, prepared using EV: LNP ratio at 3:1, were loaded with Cy5 labelled‐EGFP mRNA. NanoAnalyzer N30 instrument (NanoFCM lnc.) equipped with dual 488/640 nm lasers was employed to analyze the size and cargo loading efficiency of HEVs. EVs served as control for the experiment. Briefly, prior to analysis of samples, the instrument was calibrated using fluorescent 250 nm silica nanoparticles of known concentration. A proprietary 4‐modal silica nanosphere cocktail (S16M‐Exo, NanoFCM Inc.) was applied as size reference for generation of a size standard curve. The instrument settings were kept constant when analyzing calibration beads, size standard beads and samples. All samples were diluted using filtered PBS to attain a particle count within the optimal acquisition range of 2000–12,000/min for analysis. All samples were acquired for 60 s under sample flow control of 1.0 KPa setting. Light scattering and fluorescence signal of individual nanoparticles were collected simultaneously on single‐photon counting avalanche photodiodes detectors in the specific channels, side scatter (SSC) FF01–488/24 and red fluorescence channel FL2–670/30. Data was processed using the nFCM Professional Suite v2.0 software (NanoFCM lnc.). Data of the diluent, filtered PBS, was used for background correction. The size of samples was determined by interpolation based on the size standard curve generated. Proportional analysis of subpopulations separated by fluorescent signal was achieved.

### 2.10 Single Particle Automated Raman Trapping Analysis

LNP, EV and HEV samples were diluted in PBS and 100 µL of each sample were placed on microscopy slides then loaded in SPARTA AGIS I (SPARTA Biodiscovery) for measurement. Each sample was analyzed with an acquisition time of 10 s over ∼1.5 h period to result in approximately 300 particles being trapped for analysis. The spectra of the successfully trapped nanoparticles were pre‐processed using the defined script in the SPARTA Discovery software. The mean spectra ± standard deviation (SD). were calculated and spectra of various samples were loaded and visualized in the same plot. Raman peaks to indicate compositions of interest were assigned in the spectra and shown in Table 1 (Penders et al. 2021). Signal of identified peaks was quantified by univariant analysis using the SPARTA Discovery software. The pre‐processed Raman spectra were imported to Qlucore Omics Explorer 3.9 followed by dimensional reduction analysis PCA and t‐SNE clustering. The top 3 principal components were exported and plot in GraphPad Prism v.9.4.0.

**TABLE 1:** Composition Raman peaks assignment.

Bands	Composition/vibration	Raman shift (cm^−1^)
Ionizable lipid	MC3	1260, 1300, 1437, 1635, 1658
Polymer	PEG	850
Lipoprotein	Cholesterol	609, 698
Proteins	C–C stretch protein β‐sheet Phenylalanine	989 1004

### 2.11 Cre Reporter Assay *In* *Vitro*

An in‐house generated Cre reporter cell line with HEK293T background was implemented for Cre reporter assay *in* *vitro*. A Cre reporter cassette with sequences encoding puromycin antibiotic resistance between loxP sites and EGFP‐NanoLuc was synthesized and cloned into a pIRESneo3 vector on NheI and BamHI restriction sites. HEK293T cells were transfected with the Cre reporter vector using lipofectamine 3000 according to the manufacture's instruction. Following routine maintenance and selection with puromycin, the Cre reporter cell pool was generated. Cre reporter cells were seeded in 96‐well plate at a density of 30,000 cells per well, in complete media consisting of DMEM, Glutamax and 10% foetal bovine serum (FBS), and incubated at 37°C in a humidified incubator with 5% CO_2_. Cells were allowed to adhere overnight and treated with various doses, in particular 10, 100 and 1000 ng total RNA per well (corresponding to 0.067, 0.67 and 6.67 µg/mL), of HEVs that were loaded with Cre mRNA and produced using different EV: LNP particle ratios. Cells treated with PBS or LNPs served as negative and positive controls, respectively. Cells were maintained and imaged in Incucyte S3 Live Cell Image and Analysis System (Sartorius). Nine microscopic images per well were captured every 8 h to monitor the kinetic of HEV delivery efficiency. Image analysis was performed using Incucyte 2021A software (Sartorius). Delivery efficiency of HEVs was further quantified by flow cytometry after 72 h treatment. Cells were harvested, washed and resuspended in PBS containing 2% FBS and 50 mM EDTA for analysis on an IntelliCyt iQue Screener Plus instrument equipped with ForeCyt software (v 6.2.6752). For data analysis, negative control cells were used to gate cell population and as background for gating of EGFP positive events. Three independent experiments were conducted using three batches of samples.

### 2.12 Cell Growth and Viability Assay

To evaluate if HEV treatment affected the growth of cells, the kinetics of cell confluence, metabolic activity of cells and cell viability were analyzed. Nine microscopic images per well were captured every 8 h, and cell confluence was quantified using Incucyte 2021A software. The cell counting kit 8 (CCK8, Sigma‐Aldrich) and Zombie violet fixable viability kit (BioLegend) were applied according to the manufacturer's instructions to quantify the metabolic activity and viability of cells, respectively. Briefly, CCK8 reagent was added to the wells after 72 h treatment and incubated at 37°C for 2 h. Absorbance at 560 nm was then read in a FLUOstar Omega plate reader (BMG Labtech). Data was subtracted by background absorbance from wells with only media. To quantify cell viability post‐treatment, cells were stained with Zombie violet dye. In brief, cells were harvested, washed, resuspended in the dye dilution (1:1000) and incubated at RT for 30 min. After staining, cells were washed and resuspended in PBS with 2% FBS and 50 mM EDTA and acquired on an IntelliCyt iQue Screener Plus instrument. For the calculation of cell viability, total and viable cell counts were analyzed using ForeCyt software (v 6.2.6752). Three independent experiments were conducted using three batches of samples.

### 2.13 GAL9 Reporter Assay and Delivery of EGFP mRNA *In* *Vitro*

HEK293, Huh7, HeLa and SHSY5Y cell lines stably expressing mCherry‐Galectin9 via targeted AAVS1 integration were generated as previously described (Munson et al. 2021). Cells were seeded into 384‐well PhenoPlates (PerkinElmer: 6057300) in complete media and incubated overnight to allow adherence. Prior to dosing and imaging, Hoechst 33342 was added to the cell culture medium at 0.5 µg/mL for staining of cell nuclei. HEV formulations, particularly HEV R1 and HEV R4, were loaded with a mixture of unlabelled‐ and Cy5‐labelled EGFP mRNA following acidic fusion as described above and administered to cells at a series of doses based on RNA amount. MC3 LNP with the same mRNA payload and DiO labelled EV served as controls. For DiO labelling, EVs were mixed with 5 µM DiO Lipophilic Tracer (Invitrogen, D7778) and incubated at 37°C for 15 min. Excess dye was removed by washing with PBS, and DiO labelled‐EVs were pelleted by ultracentrifugation using a bench‐top ultracentrifuge equipped with a TLA‐55 rotor (Beckman Coulter). Particle numbers of EVs and HEVs were quantified by NTA. Dosing of DiO labelled‐EV was based on particle number, which was the same as the particle number of HEVs at the corresponding RNA amount‐based dose. Live cell imaging was carried out using a CV8000 (Yokogawa) spinning disk confocal microscope equipped with a humidified imaging chamber maintained at 37°C and 5% CO_2_. Images were taken using a 405 nm laser (BP445/45 nm), 488 nm laser (BP522/35), 561 nm laser (BP600/37) or 640 nm laser (BP676/29) for respective fluorophores with a 20× water immersion objective. Images were processed and analyzed using Columbus image‐analysis software (Perkin Elmer, v2.9.1) to identify cellular structures and quantify cellular intensities. Data were exported and processed further in Spotfire (Tibco, v11.4.0) for normalization prior to plotting in Prism (Graphpad, v9.1.0).

### 2.14 Delivery of Firefly‐Luciferase mRNA *in* *vivo*

To demonstrate if HEVs can functionally deliver mRNA *in* *vivo*, HEVs formulated with Expi293F‐EV and LNP loaded with Firefly‐luciferase (Fluc) mRNA (Trilink, L7602) using an EV:LNP particle ratio of 3:1 was prepared and dosed to C57BL/6Ncrl mice (purchased from Charles River). The animal work was performed in accordance with the National Institute of Health (NIH) guidelines for use of experimental animals and ethical approval was obtained from the Animal Ethics Committee at Gothenburg University (Gothenburg Ethical Review Board number EA 2194‐2019). Mice were anesthetized with Isoflurane and 5 mL/kg of HEVs, corresponding to 0.20 or 0.75 mg FLuc mRNA per kg animal weight, were injected intravenously. The same volume of vehicle buffer PBS was injected as a negative control. Six hours post dosing, luciferin (5 mL/kg, RediJect D‐Luciferin, PerkinElmer) was administrated subcutaneously. The whole‐body scan of the mice was performed 20 min after luciferin administration using an IVIS Spectrum (PerkinElmer). Whole blood was collected while animals were still anaesthetized from the tail vein into EDTA‐coated collection tubes (Microvette) and centrifuged at 7000 × *g* at 4°C to obtain plasma. Mice were sacrificed and organs of interest including heart, kidney, liver, lungs and spleen were dissected and IVIS scanned ex vivo to examine the expression of FLuc at organ level. The collected plasma was stored at –80°C until use for multiplexing ELISA analysis of cytokines. A panel of representative cytokines, including proinflammatory cytokines (IL‐1β, IL‐2, IL‐5, IL‐6, IL‐12p70, IFNγ and TNFα), anti‐inflammatory cytokine IL‐10, and chemokines (KC and MCP‐1), and liver enzymes, alanine transaminase (ALT) and aspartate aminotransferase (AST), were selected for evaluation of *in* *vivo* safety profile of HEV‐mRNA. The cytokines analysis was performed according to manufacturer's instructions using a Milliplex Mouse Cytokine/Chemokine Magnetic Bead 10 Plex Panel (Merck Millipore, cat. no. MCYTOMAG‐70K) and a Bio‐Plex 200 System (Bio‐Rad). Plasma ALT and AST were quantified using commercially available mouse ELISA kits (Abcam, cat. no. ab282882; Abcam, cat. no. ab263882) according to the manufacturers’ instructions.

### 2.15 Statistical Analysis

Statistical analyses were performed using GraphPad Prism v.9.4.0. Data were presented as mean ± standard error of mean (SEM), where “*n*” denotes the number of independent experiments or biological replicates. Data sets were first tested using Shapiro‐Wilk test for analysis of data normality. In cases where the data followed a normal distribution, One‐way or Two‐way ANOVA tests were used for multiple comparisons, followed by Dunnett's or Tukey's post‐hoc tests as appropriate. Kruskal‐Wallis non‐parametric test following Dunn's post‐hoc test was applied if data did not pass the normality test. For comparison of two groups, two‐tail unpaired *t* test was applied. A *p* value < 0.05 was considered as statistically significant.

## 3 Results

### 3.1 HEV Shows Successful Loading of mRNA, Incorporation of LNP Components and Preservation of EV Features

**FIGURE 1:**
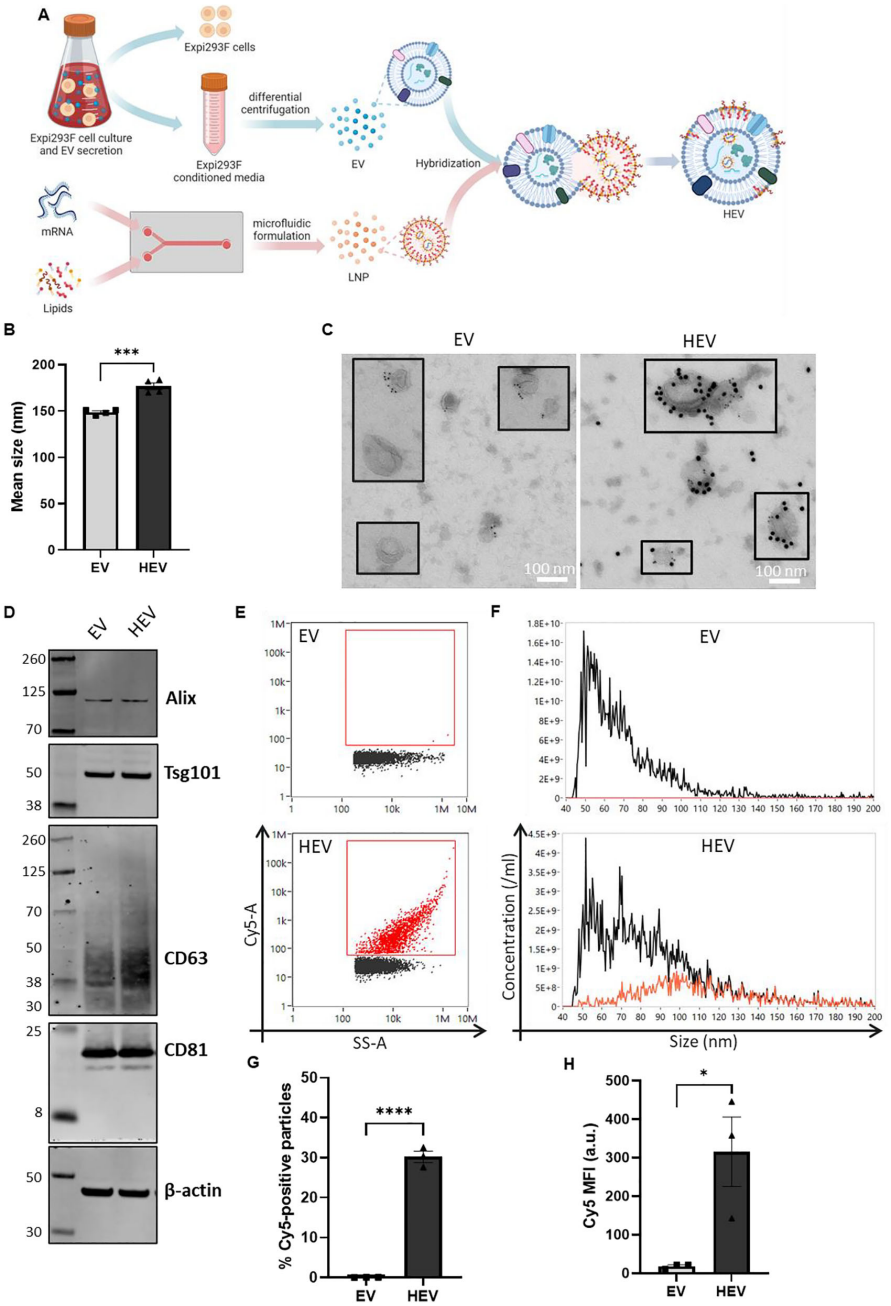
Loading of exogenous mRNA and preservation of EV features in HEV. (A) Schematic illustration of HEV production. (B) Mean size of EV and HEV measured by nanoparticle tracking analysis (NTA). (C) Representative images of immunoelectron microscopy of EV and HEV dual‐labelled with anti‐CD63, classical marker of EV and anti‐PEG, component of LNP. Detection of CD63 and PEG are indicated by presence of 6 and 15 nm gold particles, respectively. Scale bar, 100 nm. (D) Representative images of Western blot analysis of EV and HEV. Classical set of EV markers including Alix, Tsg101, CD63 and CD81 are detected. β‐actin served as sample loading control. The left lane shows ladders with different molecular weight. (E) Representative dot plots of side scatter (SS‐A) versus Cy5 intensity of EV and HEV at single particle level analyzed by nanoflow cytometry (NanoFCM). Cy5‐positive population was gated using PBS blank as control. (F) Representative size distribution plot of total population and Cy5‐positive subpopulation of particles. Black line, total population of particles; red line, Cy5‐positive subpopulation of particles. (G) Percentage of Cy5‐positive population of particles in HEV sample and native EV control. (H) Mean fluorescence intensity (MFI) of Cy5 in the total particle population of HEV sample and native EV control. All data is presented as mean ± standard error of mean (SEM), *n* = 3–4 biological replicates. *p* value determined by unpaired *t* test, * indicates statistically significant, where **** *p* value < 0.0001, *** *p* value < 0.001, * *p* value < 0.05.

In the present study, we explored a novel approach (BEER 2016) to generate HEV via incubation of EV and LNP particle mixtures at low pH conditions in an attempt to load exogenous mRNA cargo through fusion of EV particles with mRNA‐loaded LNPs (Figure 1A). Native EVs were isolated from Expi293F cell culture conditioned media by differential ultracentrifugation. EV production efficiency and purity ratio are shown in Figure S1. The ionizable cationic lipid, DLin‐MC3‐DMA (MC3), is pH responsive with an apparent pKa at 6.44 and was selected for LNP formulation in the present study (Jayaraman et al. 2012). LNPs were formulated by microfluidic mixing of lipid mixture in ethanol and mRNA in acidic aqueous buffer at the defined molar ratio of each component. Fusion of EV and LNP mixtures was induced by incubation at acidic conditions followed by ultracentrifugation to recover generated HEVs. As a proof‐of‐concept, HEVs were produced using EV: LNP at a particle ratio of 3:1, followed by analysis with complementary techniques to examine the biophysical features of HEV and the loading of exogenous mRNA.

The mean size of HEV particles (∼177 nm) increased in comparison with the source particles, EVs (∼149 nm) and LNPs (∼72 nm), as determined by NTA analysis (Figure 1B). The size increase of HEV particles may be a result of cargo loading as a similar observation was reported in a previous study showing the size of EVs increased from 120 to 140 nm post‐loading (Tsai et al. 2021). The morphology of HEVs was visualized by immunoTEM. HEVs showed similar morphologies to EVs, which exhibited spherical vesicular structures with heterogenous sizes (Figures 1C and S2B). Moreover, double immunolabeling with anti‐CD63 and anti‐PEG gold‐labelled antibodies, representing components of EV and LNP, respectively, showed EVs with either single positive of CD63 labeling or negative for both CD63 and PEG labeling (Figures 1C and S2A). While LNPs were only positive with detection of PEG (Figure S2C). HEVs by contrast displayed components of both EV and LNP. The presence of EV protein and LNP lipid components in HEVs suggested that HEV was an outcome of EV‐LNP fusion. To further confirm the preservation of EV originated protein components in HEVs, we expanded the detection of a panel of typical EV protein markers, including Alix, Tsg101, CD63 and CD81, by Western blot. Our result showed that HEVs carried all the examined EV protein markers at a similar level to native EVs (Figure 1D).

Next, we assessed if the exogenous mRNA cargo borne by the LNPs was successfully loaded into the resulting HEVs. To achieve this, we employed Nanoflow cytometry (NanoFCM) to analyze HEVs produced using LNP with 20% Cy5‐labelled EGFP mRNA spike‐in as cargo (Figure 1E–H). As shown in the representative dot plots (Figure 1E), a distinct subpopulation of particles in HEV was positive with Cy5 signal, where no Cy5 signal was detected in native EV, which served as a control, indicating successful loading of exogenous mRNA into HEVs. Overall, HEVs exhibited differential size distribution profiles in comparison with those of EVs (Figure 1F). In particular, HEVs showed an increased proportion of relatively large particles, among which the majority of identified Cy5‐positive subpopulation of HEV distributed at the relatively large size range 80–130 nm (Figure 1F). This observation may indicate cargo loading resulted in the increase of particle size, which was well in line with the increase of mean size of HEV analyzed by NTA (Figure 1B) and a previous report (Tsai et al. 2021). Nevertheless, though the same trend of increased particle size of HEVs was obtained by both NanoFCM and NTA, the absolute particle size was reported differently by NTA and NanoFCM (Figures 1B and S3). Such a difference was also reported in a previous study, where NanoFCM was shown to predominantly detect small particles (Arab et al. 2021; Andreia et al. 2021). This may be due to the different principles and detection sensitivities of the two techniques for the quantification of particle size. We further quantified the percentage of Cy5‐positive particles and mean fluorescence intensity (MFI) of Cy5 in the total population of particles as an estimation of the loading efficiency of exogenous mRNA cargo (Figure 1G,H). Across three independent batches of HEV samples, a significantly higher percentage of the HEV particles (*vs*. EV control), on average around 30%, were shown to be positive with Cy5 signal at a considerably high MFI.

**FIGURE 2:**
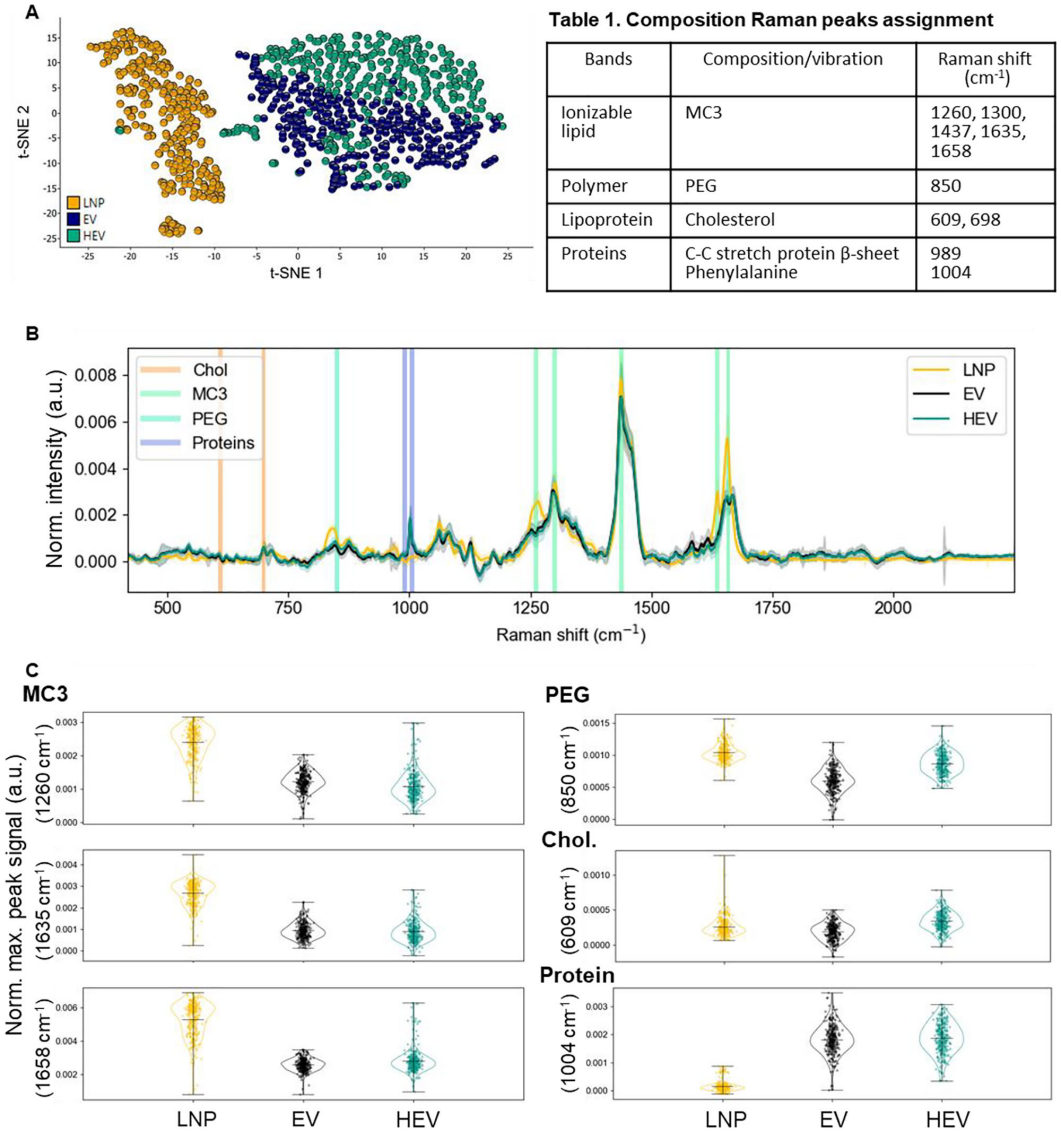
Incorporation of LNP compositions in HEV. SPARTA Raman spectra compositional analysis of LNP, EV and HEV. (A) Multivariant analysis of Raman spectra showed clustering of nanoparticles by t‐SNE plot. (B) Raman spectra signal of LNP, EV and HEV (*n* = 300, mean ± SD), spectral regions pertaining to various signals of identified compositions indicated as shown (peaks assignment showed in Table 1). (C) Quantitative comparison of identified components, including MC3 ionizable lipid, PEG, cholesterol and proteins, in LNP, EV and HEV was shown by violin plots with the median and range indicated by black line and error bar, respectively (*n* = 300).

To further explore whether components besides exogenous mRNA were incorporated from LNP to HEV, we employed single particle automated trapping analysis (SPARTA) for comprehensive examination of the overall compositions of particles in a nondestructive manner. The Raman spectra acquired via SPARTA provide a detailed fingerprint of the compositions of the analyzed nanoparticles. Comparison of the large data set of Raman spectra of all trapped single particles by dimensional reduction analysis revealed the differences in compositions across various types of nanoparticles (Figures 2A and S4A,B). Both principal component analysis (PCA) and t‐distributed stochastic neighbour embedding (t‐SNE) revealed a similar pattern, identifying three distinct clusters: LNP, EV and HEV. The HEV and EV clusters were positioned closer together compared to LNP, reflecting their similar yet distinct compositions. The top 3 PCs, as shown in Figure S4B, demonstrated the compositional variances across LNP, EV and HEV. To identify and quantify compositions of interest in HEV, comparing with their source particles, peaks pertaining to these components, based on the previous publication (Penders et al. 2021) and analysis of MC3 ionizable lipid raw material (Figure S4C), were assigned in the overlay spectra of LNP, EV and HEV (Figure 2B and Table 1). Signal of MC3 ionizable lipid‐associated peaks at 1260, 1635 and 1658 cm^−1^, PEG‐associated peak at 850 cm^−1^, cholesterol‐associated peak at 609 cm^−1^ and protein‐associated peak at 1004 cm^−1^, were quantified (Figure 2C). As shown in the violin plot, a fraction of analyzed HEV particles showed increased signal across the three peaks associated with MC3 lipid in comparison with EV particles. This result potentially indicated a fraction of HEV particles incorporated MC3 lipids from LNP. In line with the above immunoTEM observations, HEV showed increased signal of the PEG‐associated Raman peak compared with EVs. While comparing with both source particles, HEV particles showed a trend of increased cholesterol, indicated by the median signal of the analyzed particle populations. Peak associated with phenylalanine, indicating protein components were quantified, where both EV and HEV showed comparable level of signal, which was much higher than that of LNP. These results demonstrated potential incorporation of LNP compositions, including MC3 lipid, PEG and cholesterol, and preservation of protein components originated from EVs in the HEV particles. Moreover, as reflected in the clustering of Raman spectra, no traces of original unmodified LNP signals were detected in HEV samples at a single particle level.

### 3.2 Parameter Optimization to Improve HEV Production

**FIGURE 3:**
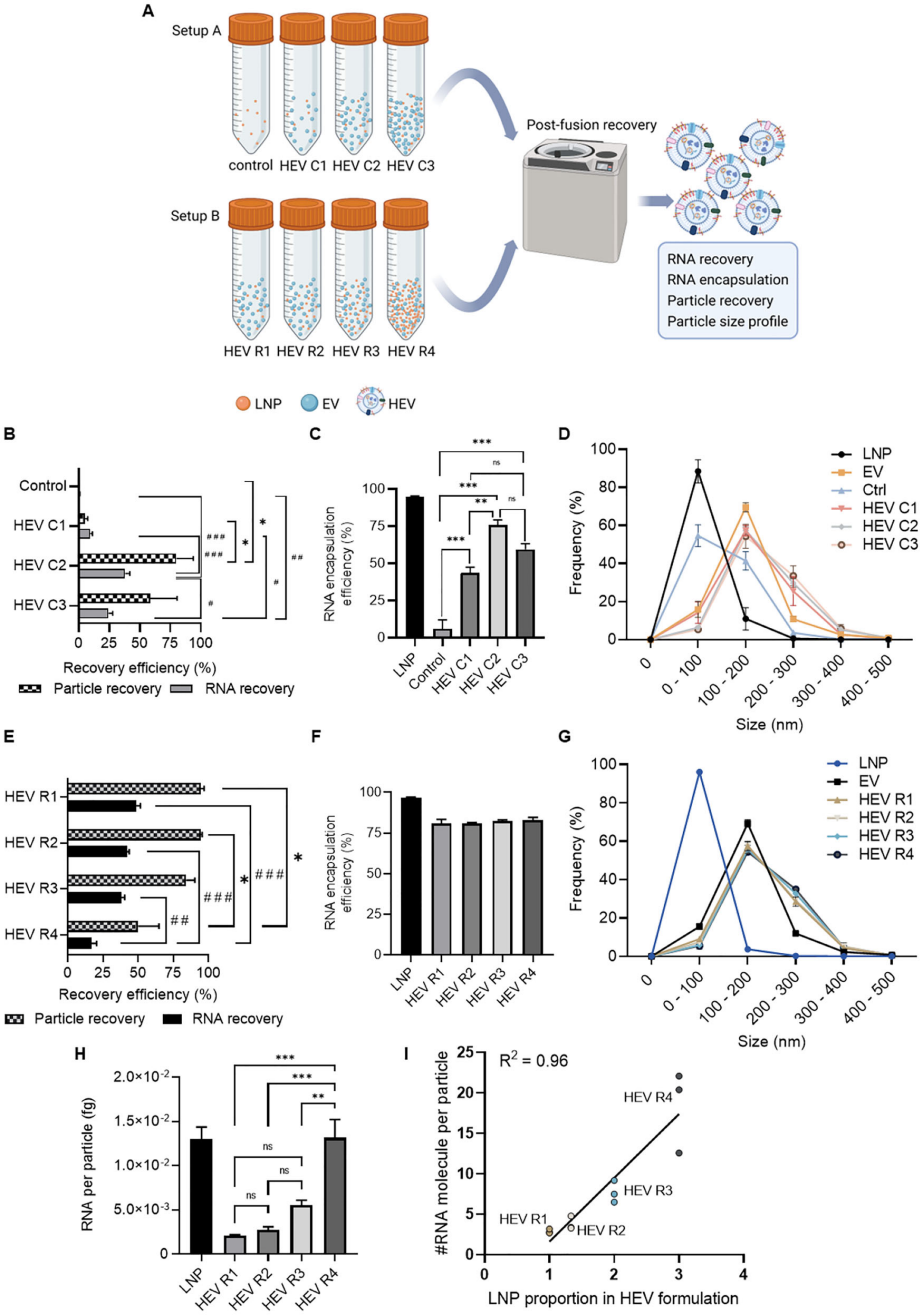
Parameter optimization of HEV production. (A) Schematic illustration shows the experimental setup of method development process for HEV production. Experiments A and B focused on optimization of particle concentration and EV: LNP particle ratio in the HEV production reactions, respectively. Experimental groups HEV C1, HEV C2 and HEV C3 represent HEV produced at particle concentrations of 10^10^, 10^11^ and 10^12^ particles/mL. Production reaction only containing LNP served as control. In setup B, HEV R1, HEV R2, HEV R3 and HEV R4 represent HEV produced at EV: LNP particle ratio ranging from 3:1, 2:1, 1:1 and 1:3, respectively. (B, E) Particle and RNA recovery efficiency of HEV produced using different concentrations of particles (setup A) or using different EV: LNP particle ratios (setup B), respectively. (C, F) RNA encapsulation efficiency of HEV produced using different concentrations of particles (setup A) or using different EV: LNP particle ratios (setup B), respectively. (D, G) Size distribution of different groups of nanoparticles in experiment setup A and setup B, respectively. Results are presented as percentage of each subpopulation of particles at the indicated size range. (H) Quantification of RNA amount in each group of nanoparticles, normalized to particle number obtained by NTA. All data is presented as mean ± SEM, *n* = 3 biological replicates. * and # indicate statistically significant, where *** and ### *p* value < 0.001, ** and ## *p* value < 0.01, * and # *p* value < 0.05. ns, non‐significant. (I) Correlation analysis of RNA loading level in different formulations of HEV particles versus the input of LNP in the corresponding HEV formulations. Dataset of converted RNA molecule number per particle was used for the analysis. Individual data points (*n* = 3), linear regression line calculated based on the mean value of each formulation and *R* ^2^ value obtained from Pearson correlation test were indicated in the graph.

Following the proof‐of‐concept of loading mRNA via EV‐LNP fusion, we further evaluated different parameters along the production process, including particle concentration and EV:LNP particle ratio, to achieve optimal recovery efficiency and cargo loading efficiency. We first examined if particle concentration influenced the production of HEV via comparison of HEVs (HEV C1, HEV C2 and HEV C3) produced using three different concentrations of particles, 10^10^, 10^11^ and 10^12^ particles/mL, respectively, but with constant EV: LNP particle ratio in the fusion reactions (Figure 3A). Cre mRNA was used as an exogenous RNA payload in these experiments. A fusion reaction merely containing the same number of LNPs alone served as a control to examine whether LNPs would be co‐isolated during recovery by ultracentrifugation (UCF). Following HEV production, the recovery efficiency of both particles and RNA, RNA encapsulation efficiency and particle size distribution were evaluated. HEV C2 yielded the highest particle and RNA recovery among all conditions (Figure 3B). The recovery efficiency of HEV C3 was slightly lower than that of HEV C2 but significantly higher than that of HEV C1 and the control group. Both HEV C1 and the control group resulted in poor recovery (Figure 3B). The very poor recovery (<1%) of both particles and RNA in the control group suggests the vast majority of unfused LNP components were eliminated post‐UCF. This may be due to the lower density of LNPs compared to EVs and thus poorly sedimented, or due to destruction of LNPs during the UCF step, as they may not resist the high‐speed of centrifugation. Such an assumption can be partially supported by recent research showing that LNPs usually possess a density of ∼1.0 g/mL (Henrickson et al. 2021), while EVs typically have a density in a range of 1.10–1.19 g/mL (Stam et al. 2021). Our results validate that the different physical properties of LNPs and EVs allowed the efficient separation of HEV from LNPs post‐fusion via an ultracentrifugation approach.

We next analyzed if particle concentration influenced the RNA encapsulation of HEV via determination of RNA amount in un‐lysed and detergent‐lysed particles. Among the three HEV groups, HEV C2 resulted in RNA encapsulation efficiency of ∼75%, whereas HEV C1 and HEV C3 showed ∼50% RNA encapsulation efficiency (Figure 3C). Notably, dramatically lower RNA encapsulation was shown in the control group where only LNPs were present in the fusion reaction and underwent incubation at acidic conditions followed by ultracentrifugation (Figure 3C). The differences of RNA encapsulation level in the presence versus the absence of EVs suggested the intravesicular loading and protection of mRNA in HEV post‐dissociation from the intact LNP. Profiling of size distribution revealed differential patterns among the different groups of particles, where HEV and EV shared a more comparable size profile with an increased proportion of subpopulation at the size range of 200–300 nm for HEVs (Figure 3D). This trend was observed among all the HEV groups, potentially suggesting size increase as a result of EV‐LNP fusion. On the other hand, LNP and particles recovered in the control group showed a similar size pattern and a major population under the size of 100 nm (Figure 3D).

Following the identification of an optimal particle concentration 10^11^ particles/mL for HEV production, we further evaluated if EV: LNP particle ratio impacts HEV production. Four fusion reactions were prepared using different EV: LNP particle ratios, particularly 3:1 (HEV R1), 2:1 (HEV R2), 1:1 (HEV R3) and 1:3 (HEV R4), respectively (Figure 3A). We first compared the recovery efficiency of the four groups of HEVs. Overall, HEV R1‐R3 showed comparable recovery efficiency. In comparison to HEV R4, HEV R1‐ R3 showed significantly higher recovery of particles and/or RNA (Figure 3E). Of note, up to 40%–50% of total RNA was recovered in most of the HEV formulations (Figure 3E). This result indicated considerably high loading efficiency of mRNA in HEVs, as compared to previously reported loading efficiency of small RNA (<0.5%–62%) and DNA/plasmids (1.75%–20%) by electroporation to EVs (reviewed elsewhere) (Rankin‐Turner et al. 2021). When comparing the size distribution profile, all groups of HEVs showed very comparable patterns that were distinct from the size profiles of source particles, LNPs and EVs (Figure 3G). Moreover, high encapsulation efficiency of RNA, ∼80%, was obtained in all groups of HEVs, which was significantly higher than that of input EVs, ∼20% (Figures 3F and S5A). As EV‐associated RNAs incorporated only a small portion of the total input RNAs in the fusion reactions for HEV production (Figure S5B), these results together suggested that the vast majority of RNA in HEVs was loaded from LNP and protected intravesicularly. Such a feature supports that HEV is a promising vehicle for mRNA delivery as protection of mRNA from degradation before reaching to the site of action is pivotal for their functional efficiency (Dowdy 2017). We further evaluated the mRNA loading capabilities in the HEV particle by normalizing the RNA amount to particle number of HEV. Interestingly, a trend of gradual increase of RNA per particle was observed among the four HEV formulations, indicating the possibility to modulate mRNA loading via EV: LNP particle ratio (Figures 3H,I and S6). Notably, HEV R4 demonstrated significantly higher amount of RNA per particle than the other three HEV formulations, which was comparable to that of the original LNP control (Figures 3H and S6). When converting the RNA amount per particle to number of Cre mRNA molecules per particle via theoretical calculation based on the molecular weight of Cre mRNA, it was estimated ∼3–18 Cre mRNA molecules are loaded in each HEV particle depending on the formulation (Figure S6).

### 3.3 HEV Shows Excellent Cell Tolerability *In* *Vitro*

**FIGURE 4:**
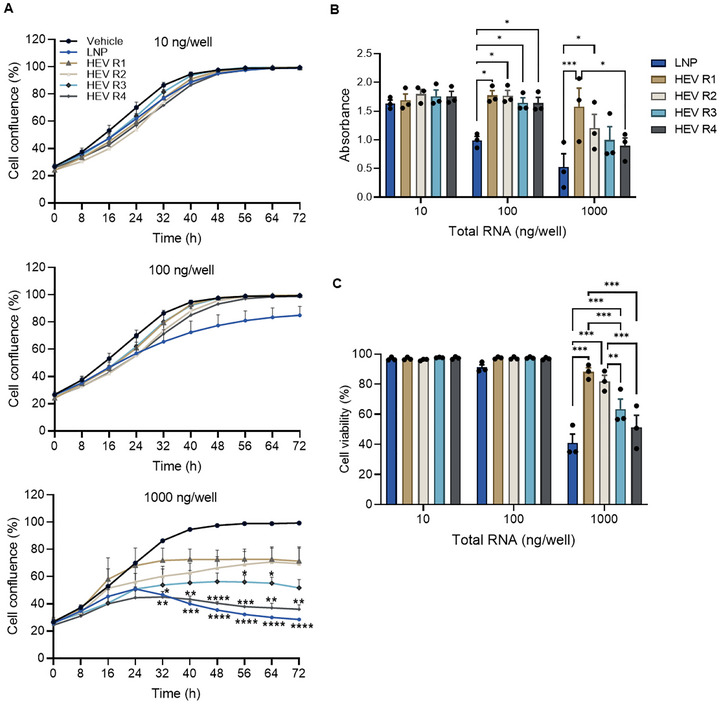
Cell tolerability of HEVs *in* *vitro*. (A) The kinetics of cell confluence upon treatment with HEV at dose of 10, 100 and 1000 ng RNA per well, respectively. (B) Total cell growth, indicated by metabolic activity of cells, upon treatment with HEVs for 72 h. Higher absorbances represent higher rates of substrate conversion and thus optimal cell growth. (C) Cell viability upon treatment with HEVs for 72 h. Cells treated with PBS buffer (vehicle) or LNP served as controls in the experiments. HEV R1, HEV R2, HEV R3 and HEV R4 represent HEV produced at EV: LNP particle ratio ranging from 3:1, 2:1, 1:1 and 1:3, respectively. All data is presented as mean ± SEM, *n* = 3 biological replicates. * indicates statistically significant by two‐way ANOVA with Dunnett's (A) or Turkey's (B, C) multiple comparisons, where *** *p* value < 0.0001, *** *p* value < 0.001, ** *p* value < 0.01, * *p* value < 0.05.

We next examined the cell tolerability of HEV when treating Cre reporter cell line with HEV formulations at three different doses, 10, 100 and 1000 ng of total RNA per well, for 72 h. Treatment with PBS or native LNPs served as controls. Cell tolerability was evaluated using complementary analysis of parameters related to cell growth, including cell confluence, cell metabolic activity and cell viability (Figure 4). Cell confluence was quantified to indicate the kinetic of cell growth under each condition over 72 h (Figure 4A). HEVs, regardless of formulations, showed no impact on the cell confluence at both low and middle doses. By contrast, LNPs showed a trend of negative impact on the cell growth post 24 h of dosing at the middle dose. Differential impact on cell growth was observed among the formulations at the high dose of treatment, where HEV R1‐R3 were better tolerated than HEV R4 and LNPs. Both LNPs and HEV R4 showed a significant reduction in cell growth post 32 h. Similar results were obtained when quantifying the NAD(P)H levels, which correspond to dehydrogenase activity, indicating metabolic activity of live cells, at 72 h post‐dosing to estimate the overall cell growth (Figure 4B). All tested HEV formulations showed comparable high absorbance at a similar level as control cells treated with PBS vehicle (∼1.5) when applying low and middle doses, indicating no impact on cell growth. However, HEV formulation‐dependent impact on cell growth was observed at the high dose, among which HEV R1 showed the best maintenance of cell growth. By contrast, treatment of LNPs resulted in a dose‐dependent decrease of absorbance, which was significantly lower than that of HEV formulations, particularly at the middle and high doses. In addition to cell growth, we harvested and stained the cells for quantification of cell viability by flow cytometry at 72 h post‐dosing (Figure 4C). Almost 100% cell viability was achieved at the low and middle doses of all tested HEV formulations, comparable with the viability of cells treated with PBS vehicle (∼97%). However, cell viability was gradually decreased when treated with the high dose of HEV R1‐R4, among which HEV R1 and HEV R2 showed better maintenance of cell viability at ∼90% compared with the other formulations (Figure 4C). In comparison, treatment with original MC3 LNP resulted in a dose‐dependent decrease of cell viability already visible at 100 ng/well on cell growth, with further detrimental effects on growth and viability at the high dose.

### 3.4 HEV Delivers Functional Cre mRNA *In* *Vitro*

**FIGURE 5:**
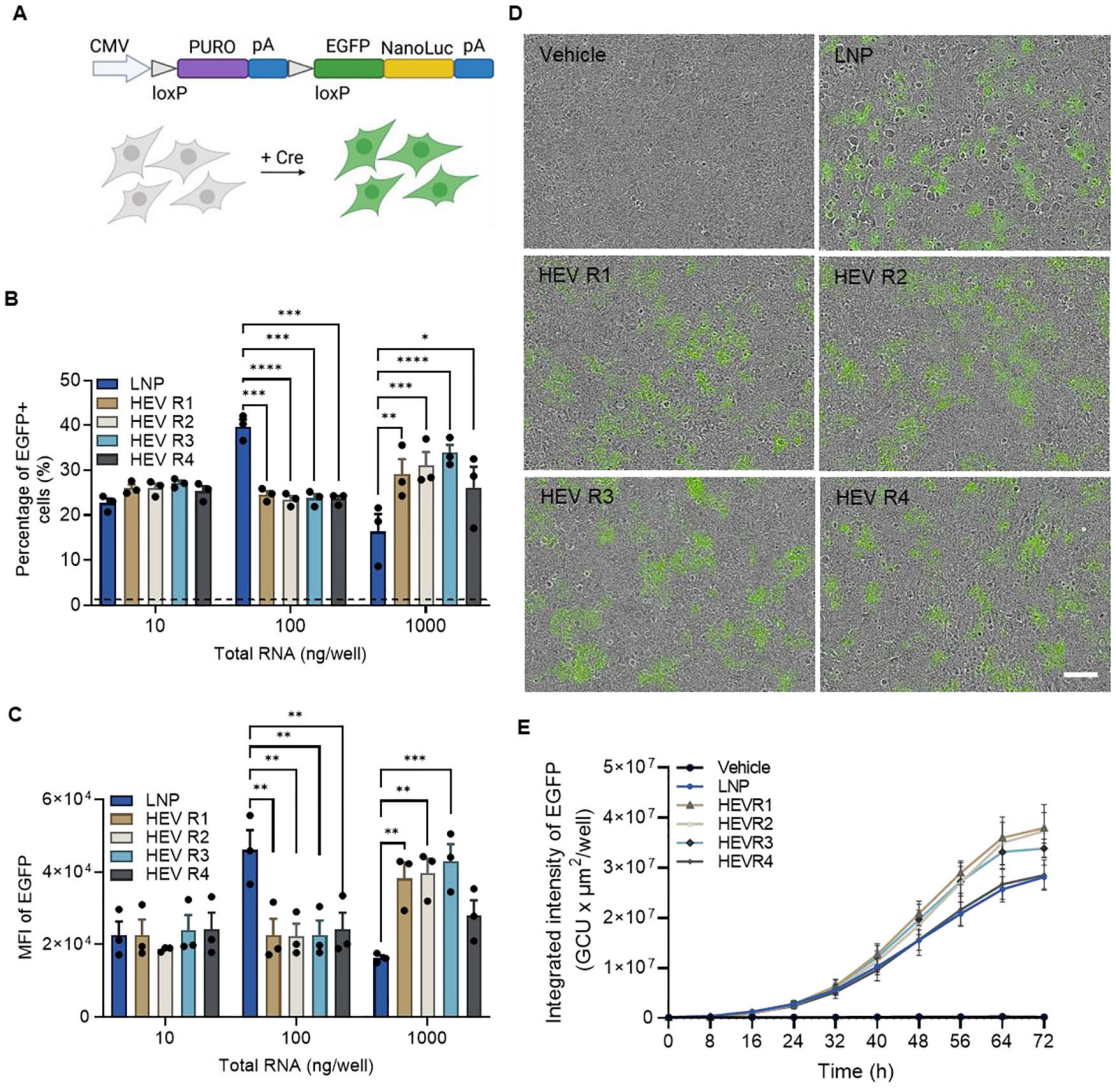
HEV delivers functional Cre mRNA *in* *vitro*. (A) Schematic illustration of Cre‐Lox reporter cassette. Cre‐Lox recombination results in switching on expression of EGFP and NanoLuc in cells, which can be directly visualized by fluorescence or indirectly quantified by luminescence assay of cell lysate. (B, C) Delivery efficiency of Cre mRNA indicated by percentage of EGFP positive cells and median fluorescence intensity (MFI) of EGFP expression in cells upon treatment of HEVs for 72 h. The dashed line represents the baseline of the vehicle control (PBS‐treated cells) and was used to establish proper gating for the EGFP‐positive cell population. (D) Representative microscopic images showed EGFP expression of cells upon HEV treatment at dose of 100 ng RNA per well for 72 h. Scale bar, 100 µm. (E) Kinetic of EGFP expression upon treatment of HEVs at dose of 100 ng RNA per well. Results were presented as total integrated fluorescence intensity of EGFP per well. LNP was served as positive control in all experiments. HEV R1‐R4 represent HEV produced at EV: LNP particle ratio ranging from 3:1, 2:1, 1:1 and 1:3, respectively. All data is presented as mean ± SEM, *n* = 3 biological replicates. * indicates statistically significant by two‐way ANOVA with Turkey's multiple comparison test, where **** *p* value < 0.0001, *** *p* value < 0.001, ** *p* value < 0.01, * *p* value < 0.05.

We next explored if mRNA loaded in HEVs can be functionally delivered using Cre mRNA as a payload and an in‐house generated Cre reporter cell model as recipient cells. Successful delivery and functional translation of Cre mRNA will result in the binding of Cre recombinase at the loxP site of the Cre‐Lox reporter cassette and subsequently switch on the expression of EGFP in the reporter cells (Figure 5A). The delivery efficiency of HEVs was indicated by quantification of EGFP expression using microscopic image‐based and flow cytometry‐based analysis. Microscopic images were taken every 8 h for quantification of EGFP expression over a 72 h period post‐dosing. Both HEVs and LNPs delivered functional Cre mRNA to switch on the EGFP expression as shown in the representative microscopic images acquired at 72 h post‐dosing (Figures 5D and S7). Quantification of the EGFP expression kinetics showed a trend that HEVs, particularly HEV R1‐R3, resulted in higher EGFP expression, especially at the low and middle doses, than HEV R4 and LNP (Figures 5E and S8). The delivery efficiency of HEVs was also determined by flow cytometry analysis of cells harvested post 72 h of treatment (Figure 5B,C). Irrespective of formulations, HEVs resulted in a similar level of EGFP expression at the low and middle doses of treatment. However, cells treated with HEV R1‐R3 showed higher percentage of EGFP positive cells and EGFP expression than that of HEV R4 at the high dose. When comparing with LNPs, HEVs showed comparable EGFP expression at the low dose and a significantly higher expression level at the high dose, except HEV R4. However, HEVs were less potent than LNP at the middle dose.

### 3.5 HEV Functionally Delivers EGFP mRNA Across Multiple Cell Lines

**FIGURE 6:**
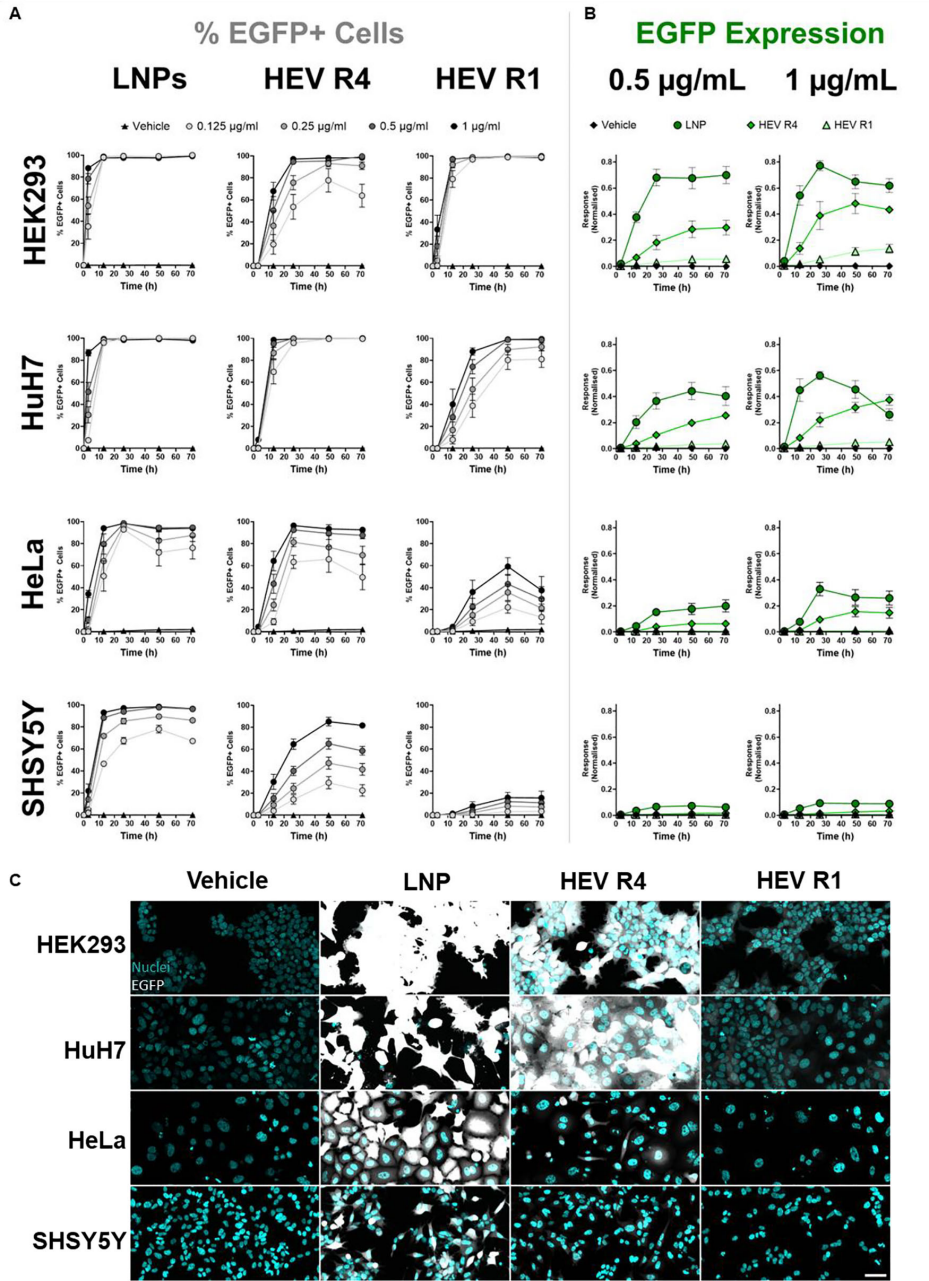
HEV functionally delivers EGFP mRNA across multiple cell lines. (A) The kinetics of transfection efficiency, as indicated by the percentage of EGFP‐positive cells, were assessed in multiple cell lines, HEK293, HuH7, HeLa and SH‐SY5Y, following administration of HEVs loaded with EGFP mRNA. (B) Delivery efficiency of EGFP mRNA in multiple cell lines, indicated by the kinetics of normalized EGFP intensity, upon administration of HEVs at the dose of 0.5 and 1.0 µg/mL. (C) Representative microscopic images showed the EGFP expression of cells upon HEV treatment at the dose of 0.5 µg/mL for 48 h. Cyan indicates nuclei and white indicates EGFP protein. Scale bar, 50 µm. LNP served as positive control in all experiments. HEV R1 and HEV R4 produced using EV: LNP particle ratio at 3:1 and 1:3, respectively were selected for examination. All data is presented as mean ± SEM, *n* = 3 biological replicates.

To further validate the functional delivery of mRNA via HEVs, EGFP mRNA was loaded in two selected HEV formulations, HEV R1 and HEV R4, and administered to four different cell lines, HEK293, HuH7, HeLa and SHSY5Y cells expressing a galectin‐9 (GAL9) reporter. The rationale to select HEV R1 and HEV R4 for further validation lies on the observation that HEV R1 and HEV R4 displayed differential phenotypic and functional features, in terms of RNA loading efficiency (Figure 2G), cell tolerability (Figure 4) and delivery efficiency of Cre mRNA (Figure 5). The percentage of EGFP positive cells were quantified over 72 h to reveal the kinetics of transfection efficiency of different cell lines upon administration of HEVs and LNP (Figure 6A). Both HEV R1 and HEV R4 resulted in cell line‐ and dose‐dependent transfection efficiency, where almost 100% transfection efficiency was achieved in HEK293 and HuH7 cell lines and less efficient transfection of HeLa and SHSY5Y cells especially by HEV R1 was observed. However, HEV R4 resulted in the pattern of transfection kinetic distinct from that of HEV R1. Particularly, HEV R4 treated cells showed earlier onset and higher transfection efficiency than that of HEV R1 at the same dose, and subsequently reached maximal transfection at an earlier time point. These results indicated that HEV R4 induced more rapid and stronger response of transfection than HEV R1. A similar result was also reflected on the level of EGFP expression as shown in Figure 6B. Cells exposed to HEV R1 and HEV R4 at the dose of 0.5 or 1.0 µg/mL showed functional translation of EGFP over 72 h, where a higher level of EGFP expression was observed in HEV R4‐treated cells across the four cell lines (Figure 6B). Correspondingly, representative microscopic images of EGFP expression across multiple cell lines at 48 h upon 0.5 µg/mL dosing were shown in Figure 6C. HEV R1‐treated cells, particularly HEK293 and HuH7, showed positive expression of EGFP compared with buffer control but a lower expression level compared with HEV R4 and LNP groups. Delivery by HEV R4 resulted in a considerably high level of EGFP expression across the four cell lines.

### 3.6 HEV Delivers mRNA Cargo Intracellularly via Induction of Endosomal Escape

**FIGURE 7:**
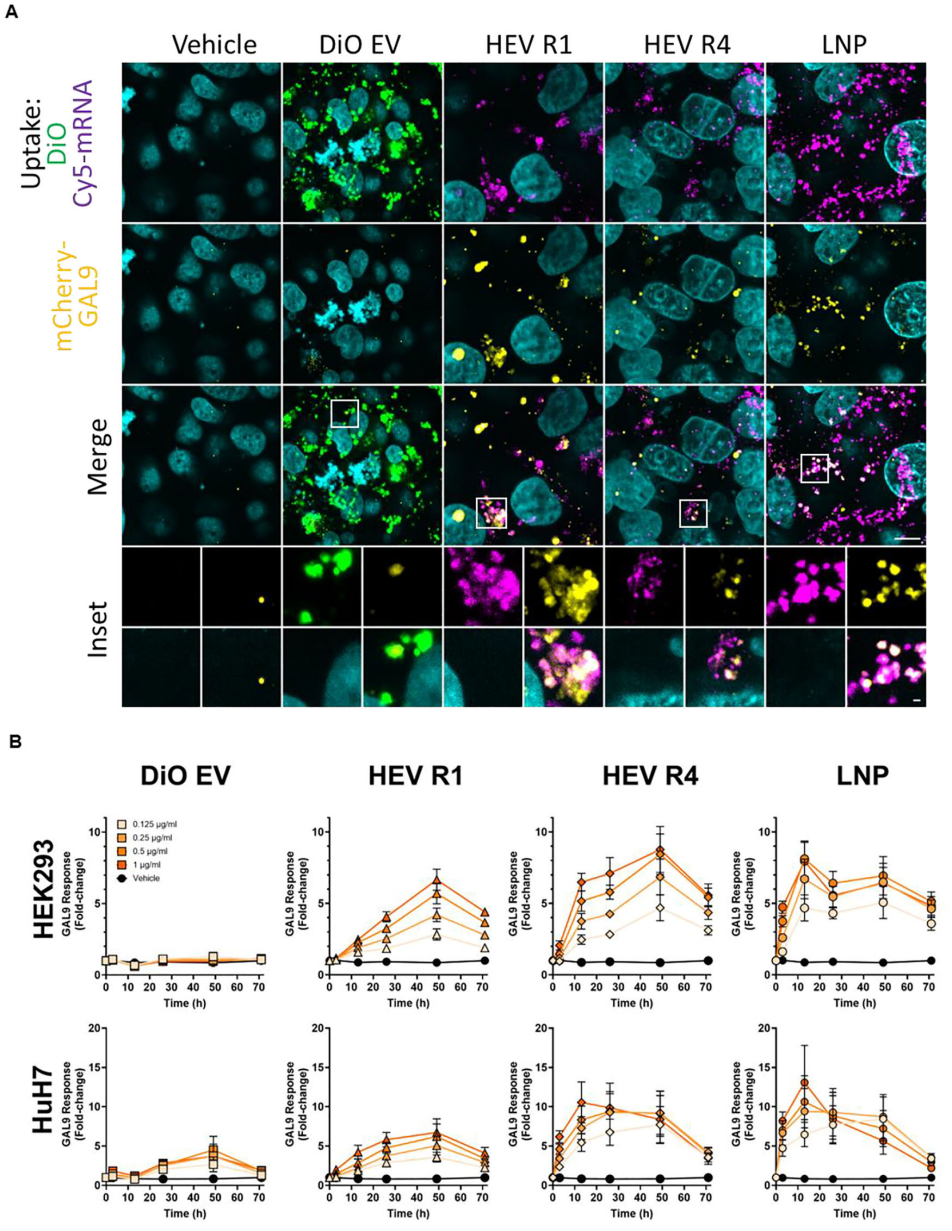
Mechanistic illustration of HEV delivery of functional cargo intracellularly. (A) Representative microscopic images showed the internalization of nanoparticles, including EV, HEV R1, HEV R4 and LNP, and correspondingly induced GAL9 response in HuH7 mCherry‐GAL9 cells at the dose of 0.5 µg/mL for 48 h. Cyan indicates nuclei, green indicates DiO labelled‐EV, purple indicates nanoparticles carrying Cy5 labelled‐EGFP mRNA and yellow indicates mCherry‐GAL9 structure. Scale bar, 10 µm; Inset, 1 µm. (B) The quantitative kinetics of GAL9 response of HEK293 and HuH7 mCherry‐GAL9 cells upon administration of nanoparticles at multiple doses for 72 h. Vehicle of PBS buffer, EV and LNP served as controls in all experiments. HEV R1 and HEV R4 produced using EV: LNP particle ratio at 3:1 and 1:3, respectively were selected for examination. All data is presented as mean ± SEM, *n* = 3 biological replicates.

We next explored the intracellular delivery mechanism of HEVs and mRNA cargo by using Cy5‐labelled mRNA for tracking of particle internalization and an mCherry‐GAL9 reporter assay to quantify endosomal escape (Munson et al. 2021). mCherry‐GAL9 is expressed diffusely in the cytosol but can be robustly recruited to endosomes upon endosomal membrane damage (Du Rietz et al. 2020; Kilchrist et al. 2019). Therefore, re‐localization and accumulation of mCherry‐GAL9 can serve as an indication of endosomal escape of internalized nanoparticles (Munson et al. 2021). HEV R1 and HEV R4 carrying Cy5‐labelled mRNA cargo were produced by fusion of native EVs and LNPs containing 20% Cy5‐labelled EGFP mRNA following the earlier‐described formulation conditions. HEVs were administered to four mCherry‐GAL9 reporter cell lines to assess their capacity for endosomal escape. LNPs and DiO‐labelled native EVs served as controls. The internalization of nanoparticles and subsequent response of endosomal escape were studied by the trafficking of Cy5‐mRNA signal or DiO signal and induction of mCherry‐GAL9 structure, respectively. The punctate signal of Cy5, DiO and mCherry‐GAL9 signal was observed as exemplified in Figure 7A for HuH7 mCherry‐GAL9 cells after 72 h of exposure at the dose of 0.5 µg/mL. HEV R1 and HEV R4 were both internalized by cells similar to LNPs and EVs, as indicated by the Cy5 or DiO puncta (Figure 7A). Interestingly, HEV R1 and HEV R4 showed induction of mCherry‐GAL9 structures similar to LNPs, which was not observed in the DiO‐EV‐exposed cells (Figure 7A). Furthermore, mCherry‐GAL9 structures were shown to co‐localize with Cy5‐mRNA signal, indicating the recruitment of GAL9 was induced by the internalized HEV nanoparticles. These results demonstrated that HEVs, irrespective of formulation, can be internalized by cells and acquire properties to induce the re‐localization of GAL9, indicative of endosomal escape.

We further explored whether the degree of GAL9 response was influenced by the different formulations of HEV and correlated with the level and exposure time of HEV by testing a range of doses of HEV R1 and HEV R4 across all reporter cell lines over 72 h. Dose‐dependent increase in induction of GAL9 response by both HEV R1 and HEV R4 was observed, as exemplified in Figure 7B for HEK293 mCherry‐GAL9 and HuH7 mCherry‐GAL9 cells. However, HEV R4 induced a stronger response of GAL9 than HEV R1 at the same dose in both reporter cell lines. Quantification of the kinetics of GAL9 response over 72 h revealed that both reporter cell lines had an earlier onset of GAL9 response upon introduction of HEV R4 than that of HEV R1, but both HEVs resulted in the maximal response of GAL9 at similar exposure time (48 h post‐dosing). These results demonstrated that HEVs induced endosomal escape via a different mode of action, which was influenced by the formulation condition. This finding was also well correlated with the observation of variable delivery efficiency of EGFP mRNA by HEV R1 and HEV R4 (Figure 6). Of note, quantitative kinetics of GAL9 punctate formation showed that DiO‐EV, regardless of dose and exposure time, resulted in no or limited induction of GAL9 response in both reporter cell lines (Figure 7B). This result suggested that EVs did not trigger endosomal escape by rupture of endosomal membranes, which was in line with the finding of a previous study using a GAL3 reporter system (Joshi et al. 2020). Joshi et al. demonstrated that EVs delivered their cargo mainly via fusion with the late endosomal membrane, rather than via induction of endosomal rupture (Joshi et al. 2020). By contrast to EVs, HEVs induced notably higher GAL9 response suggesting their in‐built capacity to induce endosomal escape is a feature likely propagated from LNP components. Nevertheless, in comparison with LNP, HEVs exposed cells exhibited delayed onset and peaking of maximal response. This may suggest the exist of different internalization routes for HEVs or mechanisms to trigger endosomal escape by HEVs versus LNPs.

### 3.7 HEV Achieves Functional Delivery of Fluc mRNA *in* *vivo* With Predominant Expression in Spleen

**FIGURE 8:**
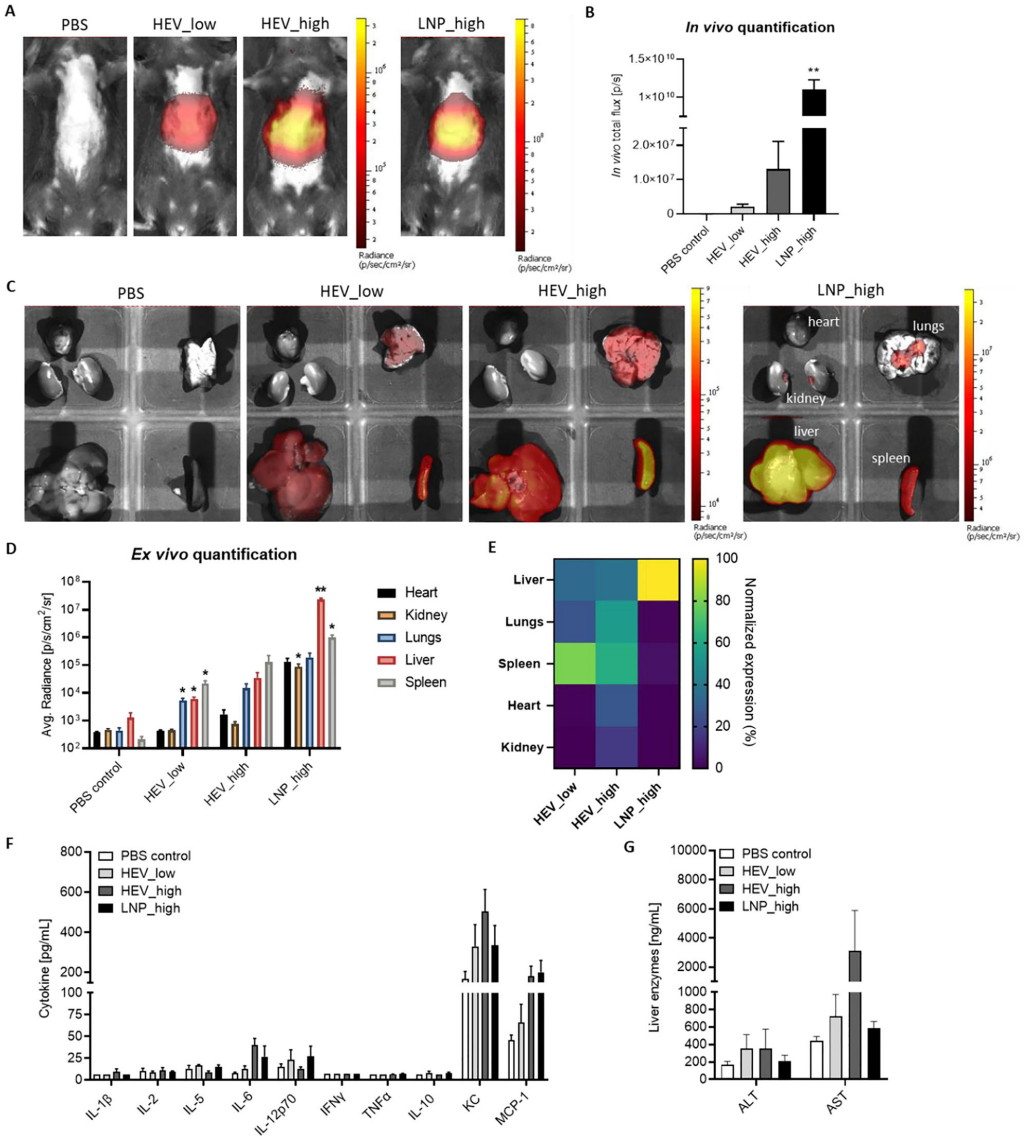
*In* *vivo* delivery of Fluc mRNA via HEV and functional biodistribution of HEV delivered Fluc mRNA. (A) Representative *in* *vivo* images of PBS‐, HEV‐ or LNP‐treated mice at 6 h post administration. Low and high indicated treatment dose at 0.20 or 0.75 mg RNA per kilogram animal weight, respectively. (B) Quantification of the *in* *vivo* imaging data. Data was shown as mean ± SEM, *n* = 5. Brown‐Forsythe and Welch One‐way ANOVA with Dunnett T3′ multiple comparison tests; **p value < 0.01 comparing with PBS control. (C) Representative *ex* *vivo* images of whole major organs, including heart, kidney, lungs, liver and spleen, excised from mice undergone different treatments. (D) Quantitative analysis of *ex* *vivo* images of major organs. Data was presented as mean ± SEM, *n* = 5. Brown‐Forsythe and Welch One‐way ANOVA with Dunnett T3′ multiple comparison tests; *p value < 0.05, **p value < 0.01 comparing with PBS control. (E) Organ biodistribution profile of HEV or LNP delivered FLuc mRNA expression. *Ex* *vivo* total flux signal of each organ was normalized to the surface area of corresponding organ and subtracted to the background signal of each organ that obtained from PBS control group. Fluc mRNA expression level of each organ was presented as normalized expression within each individual animal with the smallest signal set as 0% and the largest signal set as 100%. Heatmap was generated using mean of the normalized data within each treatment group, *n* = 5. (F) Cytokine profile of plasma collected from mice at 6 h post treatments. Data was presented as mean ± SEM (*n* = 5). Data points below the lower limit of quantification (LLOQ) were visualized at the LLOQ value. Statistical analysis revealed no significant induction of cytokines analysed. (G) Liver enzyme analysis of plasma collected from mice at 6 h post treatments. Data was presented as mean ± SEM (*n* = 5). Statistical analysis revealed no significant differences on liver enzyme levels comparing with PBS control group.

To further demonstrate functional delivery of mRNA via HEV *in* *vivo*, as proof‐of concept formulation of HEV using EV:LNP particle ratio of 3:1 was selected to load FLuc mRNA and administrated to mice at doses of 0.20 or 0.75 mg RNA per kilogram animal weight (corresponding to 1.50E + 12 or 5.46E + 12 particles per mice), respectively. Administration of the same volume of PBS vehicle served as a control for detection of background signal, while administration of LNP at the same high dose as HEV was served as positive control. Luciferin was injected subcutaneously 6 h post administration of HEV or PBS. Live imaging of the whole body was acquired and as shown in Figure 8A. HEV functionally delivered Fluc mRNA at both applied doses. Quantification of *in* *vivo* image signal revealed clear difference of signal in HEV‐ and PBS‐treated mice and dose‐dependent increase of luciferase expression level (Figure 8B). Following the live imaging, major organs were dissected and imaged *ex* *vivo* (Figure 8C). Quantification of signal at organ level revealed similar delivery pattern of HEV at both low and high doses, in which Fluc mRNA expression was majorly detected in spleen, lungs and liver (Figure 8D). Whereas signal level of heart and kidney were indifferent from background signal obtained from PBS‐treated group indicating no delivery reached these organs. Interestingly, organ biodistribution profile of HEV and LNP delivered Fluc mRNA expression was different (Figure 8E), though LNP showed higher delivery efficiency. While LNP‐delivered Fluc mRNA resulted in the vast majority of expression in liver, the highest expression of HEV‐delivered Fluc mRNA was detected in spleen, followed by the lungs and liver. This biodistribution pattern remained consistent when administrated HEV at both low and high doses. Additionally, we analyzed a panel of representative cytokines and liver enzymes in the collected plasma. Our results showed no significant induction of cytokines and liver enzymes following treatments, indicating a favorable safety profile of HEVs (Figure 8F,G). Although we did not observe a safety advantage of HEVs compared to LNPs in the present mouse study, this may be related to the dosing regimen. Expanding the dose window in future studies would be valuable for a more comprehensive assessment of HEV safety profiles comparing with other delivery systems.

## 4 Discussion

EV‐based delivery systems have attracted substantial interest owing to their intrinsic benefits, such as low immunogenicity and the capacity to cross physiological barriers, and the potential of engineering to load diverse cargoes via various approaches. However, challenges remain for efficient loading of large cargo such as mRNA in the EV‐based delivery vehicles. Hybridization of EVs with synthetic nanoparticles represents a popular approach to generate novel delivery platform with the potential to integrate the benefits of both worlds. Various approaches have been explored majorly using liposomes loading with small cargoes such as small molecule drug (Hu et al. 2021; Lv et al. 2020; Piffoux et al. 2018; Rayamajhi et al. 2019; Sun et al. 2021) or siRNA (BEER 2016; Evers et al. 2022; Ishikawa et al. 2022; Jhan et al. 2020; Zhou et al. 2022; Bader et al. 2025) as bridge vehicle and hybridization achieved by membrane extrusion (Hu et al. 2021; Rayamajhi et al. 2019; Sun et al. 2021; Evers et al. 2022; Ishikawa et al. 2022; Jhan et al. 2020; Zhou et al. 2022), freeze‐thaw cycling (Lv et al. 2020), electroporation (Jhan et al. 2020; Zhou et al. 2022) or sonication (Hu et al. 2021). Nevertheless, these hybridization methods often involved heating, dehydration or introduction of mechanical strength to the source materials during the production process, which potentially caused reduction or loss of their functions and limited to apply when having large and fragile cargoes such as mRNA (Kooijmans et al. 2013; Hood et al. 2014; Johnsen et al. 2016; Evers et al. 2022). In the present study, we explored a gentle approach for the fusion of EV‐LNP to load exogenous mRNA via incubation at acidic pH 5.5. We showed good preservation of EV features as indicated by their spheroidal morphology, similar size profile of the major particle population, expression level of typical proteins and profile of overall compositions.

Moreover, we demonstrated the successful loading of various mRNA in the produced HEVs in a relatively efficient manner, leveraging the fact that mRNA can be more easily encapsulated within LNPs. Our hybridization method development experiments revealed particle concentration and EV: LNP particle ratio as key parameters influencing HEV production and mRNA loading efficiency. HEV formulated under different EV:LNP ratios showed consistently similar size distribution pattern and high RNA encapsulation efficiency indicating reproducibility of the process. Furthermore, we observed a positive correlation of mRNA loading efficiency in HEVs and LNP proportion in the fusion reactions, indicating possibility of tuning mRNA loading in HEV via carefully adjusting the LNP proportion. Currently, a few studies have reported successful loading of mRNA in EV‐based delivery platform mostly by endogenous approaches, however, the loading efficiency was not clear or relatively low (Hung and Leonard 2016; Usman et al. 2018; Tsai et al. 2021; Kojima et al. 2018; Wang et al. 2018). For example, with the same mRNA payload, an advanced EV engineering platform showed to achieve loading of less than one Cre mRNA molecule per EV (∼0.017 Cre molecule per EV), 1000‐fold lower than our optimized formulation HEV R4 (Zickler et al. 2024). This can be due to the complexity of endogenous EV engineering platform for mRNA loading, which requires three key elements, EV sorting domain, RNA binding domain (RBD) and mRNA of interest with corresponding interacting site recognizing RBD (Hung and Leonard 2016; Kojima et al. 2018; Wang et al. 2018; Zickler et al. 2024). The three elements need to function cooperatively to be capable for successful and efficient loading of mRNA. Substantial challenges of the endogenous loading strategies include selection of EV sorting domain for active EV cargo sorting, design of RBD influencing the binding efficiency with mRNA of interest and the amount and stability of mRNA of interest transcribed in the EV producing cells. In contrast, our approach takes advantage of scalable synthetic mRNA and LNP to ensure the functional quality of mRNA and enhance the loading efficiency of mRNA. From a delivery perspective, the current approach also provides flexibility to utilize EVs from various sources, including EVs from different biofluids such as milk, which may overcome the limitation of scalable production of EVs from cell cultures. Therefore, the development of HEV offers an alternative approach for mRNA delivery via an EV‐based delivery platform.

Assessing the functional translation of loaded mRNA is essential for evaluating the effectiveness of the delivery vehicle. An interesting observation in the current study is the formulation‐ and dose‐dependent mRNA delivery efficiency and cell tolerability of HEVs. We first evaluated the delivery efficiency of Cre mRNA via multiple formulations of HEV across a broad dose range. All tested formulations showed translation of functional Cre protein, switching on the EGFP expression in reporter cells. Interestingly, HEVs showed comparable delivery efficiency at the low dose and outperformance at the high dose in comparison with LNP. This may be partially attributed to the excellent cell tolerability of HEV, which resulted in more retention of positively transfected cells. The bell‐shaped effect on the delivery efficiency of LNP is likely a consequence of a dose‐dependent increase in cytotoxicity. By contrast, HEVs propagated the excellent safety profile of EVs (Saleh et al. 2019) and demonstrated benefit to overcome dose‐limited toxicity, which is particularly valuable in therapeutic applications where expanding the dose window of mRNA therapeutics is required to achieve effective therapeutic outcomes. Therefore, the HEV delivery platform may contribute to the clinical translation of mRNA therapeutics towards a broader therapeutic area beyond vaccines (Parhiz et al. 2024). Additionally, we observed some differences in the functional features across the four HEV formulations tested, particularly at the high dose. While HEV R1‐R3 shared more similarity in cell tolerability and delivery efficiency, HEV R4 exhibited a functional phenotype closer to that of LNP. An assumption is that HEV incorporated more components from LNPs due to the increased proportion of LNP in the fusion reaction, leading to more adaptation of LNP characteristics. The observations of various amounts of RNA loading among the four HEV formulations and their gradually decreased cell tolerability at the high dose maybe partially support such an assumption. These differential functional phenotypes of HEV motivated us to further investigate two selected formulations, HEV R1 and HEV R4, with EGFP mRNA as loading cargo and expand the examination across multiple cell lines. Both HEVs showed efficient transfection of cells, resulting in functional translation of EGFP, but displayed a differential mode of action. In comparison with HEV R1, HEV R4 triggered a faster and stronger response of transfection and a higher expression level of EGFP. This trend is consistent across the tested doses and in multiple cell lines. These observations drove us to further understand the underlying mechanism of HEV‐mediated intracellular delivery of mRNA.

Endosomal escape remains a significant challenge for intracellular delivery of cargoes. Recent studies reported the inefficient delivery of EV cargo intracellularly, with only up to 30% of the internalized EVs capable of content delivery (Emeline Bonsergent et al. 2021), and prolonged retention of internalized EV in the endosomal compartment in a non‐functional manner, which subsequently leads to degradation or re‐release of EV contents (O'Brien et al. 2022). These findings highlighted that triggering endosomal escape of internalized EVs in a timely manner is critical to improve the functional delivery efficiency of EV cargoes and unlock the full potential of EV as a delivery vehicle. EVs were previously shown not to induce endosomal rupture but delivered cargoes via membrane fusion with late endosomes (Joshi et al. 2020). Aligned with this finding, we did not observe EV‐induced GAL9 translocation, indicating no endosomal membrane remodeling occurred. However, upon EV hybridized with LNP, GAL9 response was detected, suggesting an in‐built endosomal escape capacity was acquired in HEVs. Interestingly, the mode of action of HEV‐induced endosomal escape was well correlated to that of HEV‐mediated EGFP mRNA delivery efficiency. These results may suggest that the mechanism of HEV‐mediated mRNA cargo delivery was at least partially attributed to this acquired capacity to remodel the endosomal membrane and release mRNA cargo to achieve functional delivery. It was proposed that ionizable lipids become protonated and positively charged in the acidic endosomal compartment, enabling their interaction with negatively charged endosomal membrane surface to facilitate the cytosolic release of cargoes (Maugeri et al. 2019). Notably, we observed a fraction of HEV particles with incorporation of MC3 ionizable lipid based on Raman spectroscopic analysis, which may contribute to the HEV induced endosomal membrane remodeling. Additionally, Raman spectral analysis revealed a trend of increased cholesterol level in HEV particles comparing with source EVs. Cholesterol enrichment in exosomes has been recently demonstrated to promote membrane fusion and the cytosolic delivery of siRNA (Zhuo et al. 2024), highlighting its positive role in cargo delivery efficiency. Therefore, the incorporation of LNP components may explain the unique features of HEVs compared to EVs and contribute to the functional delivery of mRNA via HEV.

We further showed the functional delivery of Fluc mRNA via HEV *in* *vivo*, and another interesting finding is the favoured delivery towards the spleen by HEV versus dominant delivery to the liver by LNP. Functional biodistribution of nanoparticle‐based drug to the right tissue is crucial to achieve therapeutic effects and minimize off‐target effects. However, extrahepatic delivery is currently one of the major bottlenecks hindering the broad application of nanoparticle‐based drug delivery systems such as LNP. It is not yet fully elucidated that the mechanistic factors of nanoparticle biodistribution. However, recent studies showed administration route and particle size influence the biodistribution of LNP (Chen et al. 2016; Di et al. 2022). Small‐sized LNP (<100 nm) predominantly accumulated and delivered mRNA cargo to the liver, which was consistent with what we observed here. An optimized LNP (SORT LNP) with the addition of a fifth negatively charged lipid component resulted in a particle size of ∼200 nm achieved spleen targeting delivery, and in situ generation of CAR T cells (Álvarez‐Benedicto et al. 2023; Vaidya et al. 2024). Similarly, an engineered EV loaded with reporter fusion protein resulting in a major population of particles at size ∼177 nm displayed a predominant biodistribution in spleen (Lai et al. 2014). These findings may explain the particle size‐driven differential patterns of functional biodistribution of HEV versus LNP in the present study. Secondly, the spleen‐enriched delivery of HEV may be an outcome of the changes on their surface feature as hybridization of EVs with LNP. A previous study reported the surface protein profile of EVs, determined their distribution fate *in* *vivo* (Hoshino et al. 2015). Engineered EVs with glycosylation showed altered distribution to lungs, potentially due to the changes of the glycome on the EV surface (Royo et al. 2019). As we observed the distinct clustering of the Raman spectrum of HEV versus source EV, likely HEV displayed compositions inherited from LNP on its surface and thus altered their interaction with cells and subsequent biodistribution. Moreover, the changes in surface features of HEV may also impact their interaction with plasma proteins, leading to various profiles of protein corona formation. Protein corona has been demonstrated to be one of the key mechanistic factors driving organ‐targeting delivery of nanoparticles (Dilliard et al. 2021). Recent study showed EVs also carried protein corona, which impacts the biodistribution and cellular uptake of EVs (Liam‐Or et al. 2024). Therefore, it would be of interest to further explore the underlying mechanistic factors of HEV‐mediated functional biodistribution and how we can control these properties to achieve organ‐targeting delivery by HEV. Additionally, incorporating fluorescent labelling of EVs, LNPs and HEVs in future studies would enable tracking of particle biodistribution and facilitate direct side‐by‐side comparisons, thereby deepening our mechanistic understanding of both their distribution patterns and cargo delivery profiles. Nevertheless, the *in* *vivo* delivery efficiency of HEV was lower than that of LNP in the present study as observed in the *in* *vitro* experiments of the same formulation conditions. This may be attributed to the technical challenge of purification of HEV during the production process. In the current development, we showed elimination of LNPs from HEVs post EV‐LNP fusion and clear differences in cellular treatment with HEVs versus source EVs. However, we cannot discount potential synergistic effects with the unfused population of EVs co‐isolated in the final HEV product. Further exploration of techniques for purification of the cargo positively loaded fraction will enable obtaining the bona fide HEVs for more accurate evaluation of the cargo delivery efficiency of HEVs. Secondly, the *in* *vivo* delivery efficiency of HEV may be formulation dependent, as indicated by *in* *vitro* results. In the present study, though we selected HEV formulation with EV: LNP particle ratio 3:1 for *in* *vivo* proof of concept study, it is of interest to determine the delivery efficiency of other HEV formulations *in* *vivo* in a future study. Additionally, it is worth examining the spatial delivery of HEVs in the spleen and determining which specific cell types are targeted for mRNA delivery byHEV in future studies. Moreover, to facilitate further translational application of HEVs, it is critical to demonstrate the stability of HEV in future studies.

In conclusion, we developed an EV‐based delivery platform, HEVs, through EV‐LNP fusion, which enables efficient loading and functional delivery of mRNA. We demonstrated well‐preserved features of EVs while acquiring beneficial features from LNPs in HEVs. HEVs exhibited excellent cell tolerability and successfully delivered various mRNA across multiple cell lines. Moreover, HEV showed functional delivery of mRNA *in* *vivo* with predominant functional biodistribution to the spleen. Mechanistically, the intracellular delivery of mRNA and altered functional biodistribution by HEVs may partially be attributed to their inherent compositions from LNP, which resulted in changes in surface properties and subsequently confer the capacity to remodel endosomal membrane *in* *vitro* and alter spatial interactions in the *in* *vivo* environment.

## Author Contributions

**Xiaoqin Wang**: conceptualization, methodology, writing–original draft, writing–review and editing, visualization, investigation, validation, formal analysis, data curation, resources, software, project administration, supervision. **Michael J. Munson**: data curation, investigation, visualization, formal analysis, writing–review and editing, software. **Kristina Friis**: investigation, writing–review and editing. **Anna Marzeda**: data curation, investigation, writing–review and editing. **Andreia M. Silva**: writing–review and editing, methodology. **Franziska Kohl**: investigation, writing–review and editing. **Leif Hultin**: data curation, investigation, resources, writing–review and editing. **Raymond M. Schiffelers**: writing–review and editing, funding acquisition, resources. **Niek Dekker**: funding acquisition, writing–review and editing, conceptualization, resources, project administration, supervision.

## Conflicts of Interest

X.W., M.J.M., K.F., A.M., A.M.S., F.K., L.H. and N.D. are or were employees of AstraZeneca. All remaining authors declare no competing interests.

## Supporting information




**Supplementary Material**: jev270201‐sup‐0001‐SuppMat.docx

## Data Availability

The data that support the findings of this study are available from the corresponding author upon reasonable request.

## References

[jev270201-bib-0001] Álvarez‐Benedicto, E. , Z. Tian , S. Chatterjee , et al. 2023. “Spleen SORT LNP Generated In Situ CAR T Cells Extend Survival in a Mouse Model of Lymphoreplete B Cell Lymphoma.” Angewandte Chemie (International ed in English) 62, no. 44: e202310395. 37651468 10.1002/anie.202310395 PMC10826899

[jev270201-bib-0002] Andreia, M. , E. L.‐I. Silva , A. Gunnarsson , et al. 2021. “Quantification of Protein Cargo Loading Into Engineered Extracellular Vesicles at Single‐Vesicle and Single‐Molecule Resolution.” Journal of Extracellular Vesicles 10, no. 10: e12130. 10.1002/jev2.12130. 34377376 PMC8329990

[jev270201-bib-0003] Arab, T. , E. R. Mallick , Y. Huang , et al. 2021. “Characterization of Extracellular Vesicles and Synthetic Nanoparticles With Four Orthogonal Single‐Particle Analysis Platforms.” Journal of Extracellular Vesicles 10, no. 6: e12079. 33850608 10.1002/jev2.12079 PMC8023330

[jev270201-bib-0004] Bader, J. , P. Rüedi , V. Mantella , et al. 2025. “Loading of Extracellular Vesicles With Nucleic Acids via Hybridization With Non‐Lamellar Liquid Crystalline Lipid Nanoparticles.” Advanced Science (Weinheim) 12, no. 8: e2404860. 10.1002/advs.202404860 PMC11848734 39741121

[jev270201-bib-0005] Beer, J. D. 2016. Hybridosomes, Compositions Comprising the Same, Processes for Their Production and Uses Thereof. Anjarium Biosciences AG. https://patents.google.com/patent/US20160354313A1/en.

[jev270201-bib-0006] Bollini, S. , A. M. Smits , C. Balbi , E. Lazzarini , and P. Ameri . 2018. “Triggering Endogenous Cardiac Repair and Regeneration via Extracellular Vesicle‐Mediated Communication.” Frontiers in Physiology 9: 1497. 30405446 10.3389/fphys.2018.01497 PMC6206049

[jev270201-bib-0007] Chen, S. , Y. Y. C. Tam , P. J. C. Lin , M. M. H. Sung , Y. K. Tam , and P. R. Cullis . 2016. “Influence of Particle Size on the In Vivo Potency of Lipid Nanoparticle Formulations of siRNA.” Journal of Controlled Release 235: 236–244. 27238441 10.1016/j.jconrel.2016.05.059

[jev270201-bib-0008] Di, J. , Z. Du , K. Wu , et al. 2022. “Biodistribution and Non‐Linear Gene Expression of mRNA LNPs Affected by Delivery Route and Particle Size.” Pharmaceutical Research 39, no. 1: 105–114. 35080707 10.1007/s11095-022-03166-5 PMC8791091

[jev270201-bib-0009] Dilliard, S. A. , Q. Cheng , and D. J. Siegwart . 2021. “On the Mechanism of Tissue‐Specific mRNA Delivery by Selective Organ Targeting Nanoparticles.” Proceedings of the National Academy of Sciences of the United States of America 118, no. 52: e2109256118. 34933999 10.1073/pnas.2109256118 PMC8719871

[jev270201-bib-0010] Dosil, S. G. , S. Lopez‐Cobo , A. Rodriguez‐Galan , et al. 2022. “Natural Killer (NK) Cell‐Derived Extracellular‐Vesicle Shuttled MicroRNAs Control T Cell Responses.” Elife 11: e76319. 10.7554/eLife.76319. 35904241 PMC9366747

[jev270201-bib-0011] Dowdy, S. F. 2017. “Overcoming Cellular Barriers for RNA Therapeutics.” Nature Biotechnology 35, no. 3: 222–229. 10.1038/nbt.3802 28244992

[jev270201-bib-0012] Du Rietz, H. , H. Hedlund , S. Wilhelmson , P. Nordenfelt , and A. Wittrup . 2020. “Imaging Small Molecule‐induced Endosomal Escape of siRNA.” Nature Communications 11, no. 1: 1809. 10.1038/s41467-020-15300-1 PMC7156650 32286269

[jev270201-bib-0013] Ekström, K. , O. Omar , C. Granéli , X. Wang , F. Vazirisani , and P. Thomsen . 2013. “Monocyte Exosomes Stimulate the Osteogenic Gene Expression of Mesenchymal Stem Cells.” PLoS ONE 8, no. 9: e75227. 24058665 10.1371/journal.pone.0075227 PMC3776724

[jev270201-bib-0014] Emeline Bonsergent, E. G. , J. Buchrieser , O. Schwartz , C. Théry , and G. Lavieu . 2021. “Quantitative Characterization of Extracellular Vesicle Uptake and Content Delivery Within Mammalian Cells.” Nature Communications 12: 1864. 10.1038/s41467-021-22126-y. PMC7994380 33767144

[jev270201-bib-0015] Evers, M. J. W. , S. I. van de Wakker , E. M. de Groot , et al. 2022. “Functional siRNA Delivery by Extracellular Vesicle‐Liposome Hybrid Nanoparticles.” Advanced Healthcare Materials 11, no. 5: e2101202. 34382360 10.1002/adhm.202101202 PMC11468224

[jev270201-bib-0016] Faksova, K. , D. Walsh , Y. Jiang , et al. 2024. “COVID‐19 Vaccines and Adverse Events of Special Interest: A Multinational Global Vaccine Data Network (GVDN) Cohort Study of 99 Million Vaccinated Individuals.” Vaccine 42, no. 9: 2200–2211. 38350768 10.1016/j.vaccine.2024.01.100

[jev270201-bib-0017] Gomzikova, M. O. , V. James , and A. A. Rizvanov . 2019. “Therapeutic Application of Mesenchymal Stem Cells Derived Extracellular Vesicles for Immunomodulation.” Frontiers in Immunology 10: 2663. 31849929 10.3389/fimmu.2019.02663 PMC6889906

[jev270201-bib-0018] Gupta, D. , A. M. Zickler , and S. El Andaloussi . 2021. “Dosing Extracellular Vesicles.” Advanced Drug Delivery Reviews 178: 113961. 34481030 10.1016/j.addr.2021.113961

[jev270201-bib-0019] Henrickson, A. , J. A. Kulkarni , J. Zaifman , G. E. Gorbet , P. R. Cullis , and B. Demeler . 2021. “Density Matching Multi‐Wavelength Analytical Ultracentrifugation to Measure Drug Loading of Lipid Nanoparticle Formulations.” ACS Nano 15, no. 3: 5068–5076. 33617224 10.1021/acsnano.0c10069

[jev270201-bib-0020] Hood, J. L. , M. J. Scott , and S. A. Wickline . 2014. “Maximizing Exosome Colloidal Stability Following Electroporation.” Analytical Biochemistry 448: 41–49. 24333249 10.1016/j.ab.2013.12.001 PMC3954633

[jev270201-bib-0021] Hoshino, A. , B. Costa‐Silva , T.‐L. Shen , et al. 2015. “Tumour Exosome Integrins Determine Organotropic Metastasis.” Nature 527, no. 7578: 329–335. 26524530 10.1038/nature15756 PMC4788391

[jev270201-bib-0022] Hu, M. , J. Zhang , L. Kong , et al. 2021. “Immunogenic Hybrid Nanovesicles of Liposomes and Tumor‐Derived Nanovesicles for Cancer Immunochemotherapy.” ACS Nano 15, no. 2: 3123–3138. 33470095 10.1021/acsnano.0c09681

[jev270201-bib-0023] Hung, M. E. , and J. N. Leonard . 2016. “A Platform for Actively Loading Cargo RNA to Elucidate Limiting Steps in EV‐Mediated Delivery.” Journal of Extracellular Vesicles 5: 31027. 27189348 10.3402/jev.v5.31027 PMC4870355

[jev270201-bib-0024] Ishikawa, R. , S. Yoshida , S.‐I. Sawada , Y. Sasaki , and K. Akiyoshi . 2022. “Fusogenic Hybrid Extracellular Vesicles With PD‐1 Membrane Proteins for the Cytosolic Delivery of Cargos.” Cancers (Basel) 14, no. 11: 2635. 35681615 10.3390/cancers14112635 PMC9179877

[jev270201-bib-0025] Jayaraman, M. , S. M. Ansell , B. L. Mui , et al. 2012. “Maximizing the Potency of siRNA Lipid Nanoparticles for Hepatic Gene Silencing In Vivo.” Angewandte Chemie (International ed in English) 51, no. 34: 8529–8533. 22782619 10.1002/anie.201203263 PMC3470698

[jev270201-bib-0026] Jhan, Y.‐Y. , D. Prasca‐Chamorro , G. Palou Zuniga , et al. 2020. “Engineered Extracellular Vesicles With Synthetic Lipids via Membrane Fusion to Establish Efficient Gene Delivery.” International Journal of Pharmaceutics 573: 118802. 31715354 10.1016/j.ijpharm.2019.118802

[jev270201-bib-0027] Johnsen, K. B. , J. M. Gudbergsson , M. N. Skov , et al. 2016. “Evaluation of Electroporation‐Induced Adverse Effects on Adipose‐Derived Stem Cell Exosomes.” Cytotechnology 68, no. 5: 2125–2138. 26856590 10.1007/s10616-016-9952-7 PMC5023584

[jev270201-bib-0028] Joshi, B. S. , M. A. de Beer , B. N. G. Giepmans , and I. S. Zuhorn . 2020. “Endocytosis of Extracellular Vesicles and Release of Their Cargo From Endosomes.” ACS Nano 14, no. 4: 4444–4455. 32282185 10.1021/acsnano.9b10033 PMC7199215

[jev270201-bib-0029] Kalluri, R. , and V. S. LeBleu . 2020. “The Biology, Function, and Biomedical Applications of Exosomes.” Science 367, no. 6478: eaau6977. 32029601 10.1126/science.aau6977 PMC7717626

[jev270201-bib-0030] Kanada, M. , M. H. Bachmann , J. W. Hardy , et al. 2015. “Differential Fates of Biomolecules Delivered to Target Cells via Extracellular Vesicles.” Proceedings of the National Academy of Sciences of the United States of America 112, no. 12: E1433–E1442. 25713383 10.1073/pnas.1418401112 PMC4378439

[jev270201-bib-0031] Kilchrist, K. V. , S. C. Dimobi , M. A. Jackson , et al. 2019. “Gal8 Visualization of Endosome Disruption Predicts Carrier‐Mediated Biologic Drug Intracellular Bioavailability.” ACS Nano 13, no. 2: 1136–1152. 30629431 10.1021/acsnano.8b05482 PMC6995262

[jev270201-bib-0032] Kojima, R. , D. Bojar , G. Rizzi , et al. 2018. “Designer Exosomes Produced by Implanted Cells Intracerebrally Deliver Therapeutic Cargo for Parkinson's Disease Treatment.” Nature Communications 9, no. 1: 1305. 10.1038/s41467-018-03733-8 PMC5880805 29610454

[jev270201-bib-0033] Kooijmans, S. A. A. , S. Stremersch , K. Braeckmans , et al. 2013. “Electroporation‐Induced siRNA Precipitation Obscures the Efficiency of siRNA Loading Into Extracellular Vesicles.” Journal of Controlled Release 172, no. 1: 229–238. 23994516 10.1016/j.jconrel.2013.08.014

[jev270201-bib-0034] Krämer‐Albers, E. M. 2022. “Extracellular Vesicles at CNS Barriers: Mode of Action.” Current Opinion in Neurobiology 75: 102569. 35667340 10.1016/j.conb.2022.102569

[jev270201-bib-0035] Lai, C. P. , O. Mardini , M. Ericsson , et al. 2014. “Dynamic Biodistribution of Extracellular Vesicles In Vivo Using a Multimodal Imaging Reporter.” ACS Nano 8, no. 1: 483–494. 24383518 10.1021/nn404945r PMC3934350

[jev270201-bib-0036] Lamichhane, T. N. , A. Jeyaram , D. B. Patel , et al. 2016. “Oncogene Knockdown via Active Loading of Small RNAs Into Extracellular Vesicles by Sonication.” Cellular and Molecular Bioengineering 9, no. 3: 315–324. 27800035 10.1007/s12195-016-0457-4 PMC5084850

[jev270201-bib-0037] Lamichhane, T. N. , R. S. Raiker , and S. M. Jay . 2015. “Exogenous DNA Loading Into Extracellular Vesicles via Electroporation Is Size‐Dependent and Enables Limited Gene Delivery.” Molecular Pharmaceutics 12, no. 10: 3650–3657. 26376343 10.1021/acs.molpharmaceut.5b00364 PMC4826735

[jev270201-bib-0038] Liam‐Or, R. , F. N. Faruqu , A. Walters , et al. 2024. “Cellular Uptake and In Vivo Distribution of Mesenchymal‐Stem‐Cell‐Derived Extracellular Vesicles Are Protein Corona Dependent.” Nature Nanotechnology 19, no. 6: 846–855. 10.1038/s41565-023-01585-y PMC11186763 38366223

[jev270201-bib-0039] Lv, Q. , L. Cheng , Y. Lu , et al. 2020. “Thermosensitive Exosome‐Liposome Hybrid Nanoparticle‐Mediated Chemoimmunotherapy for Improved Treatment of Metastatic Peritoneal Cancer.” Advanced Science (Weinheim) 7, no. 18: 2000515. 10.1002/advs.202000515 PMC7509655 32999828

[jev270201-bib-0040] Massaro, C. , W. Min , D. M. Pegtel , and S. R. Baglio . 2021. “Harnessing EV Communication to Restore Antitumor Immunity.” Advanced Drug Delivery Reviews 176: 113838. 34144088 10.1016/j.addr.2021.113838

[jev270201-bib-0041] Mathieu, M. , L. Martin‐Jaular , G. Lavieu , and C. Théry . 2019. “Specificities of Secretion and Uptake of Exosomes and Other Extracellular Vesicles for Cell‐to‐Cell Communication.” Nature Cell Biology 21, no. 1: 9–17. 30602770 10.1038/s41556-018-0250-9

[jev270201-bib-0042] Maugeri, M. , M. Nawaz , A. Papadimitriou , et al. 2019. “Linkage Between Endosomal Escape of LNP‐mRNA and Loading Into EVs for Transport to Other Cells.” Nature Communications 10, no. 1: 4333. 10.1038/s41467-019-12275-6 PMC6760118 31551417

[jev270201-bib-0043] Mulcahy, L. A. , R. C. Pink , and D. R. Carter . 2014. “Routes and Mechanisms of Extracellular Vesicle Uptake.” Journal of Extracellular Vesicles 3: 24641. 10.3402/jev.v3.24641. PMC4122821 25143819

[jev270201-bib-0044] Munson, M. J. , G. O'Driscoll , A. M. Silva , et al. 2021. “A High‐Throughput Galectin‐9 Imaging Assay for Quantifying Nanoparticle Uptake, Endosomal Escape and Functional RNA Delivery.” Communications Biology 4, no. 1: 211. 33594247 10.1038/s42003-021-01728-8 PMC7887203

[jev270201-bib-0045] O'Brien, K. , S. Ughetto , S. Mahjoum , A. V. Nair , and X. O. Breakefield . 2022. “Uptake, Functionality, and Re‐Release of Extracellular Vesicle‐Encapsulated Cargo.” Cell Reports 39, no. 2: 110651. 35417683 10.1016/j.celrep.2022.110651 PMC9074118

[jev270201-bib-0046] Parhiz, H. , E. N. Atochina‐Vasserman , and D. Weissman . 2024. “mRNA‐Based Therapeutics: Looking Beyond COVID‐19 Vaccines.” Lancet 403, no. 10432: 1192–1204. 38461842 10.1016/S0140-6736(23)02444-3

[jev270201-bib-0047] Penders, J. , A. Nagelkerke , E. M. Cunnane , et al. 2021. “Single Particle Automated Raman Trapping Analysis of Breast Cancer Cell‐Derived Extracellular Vesicles as Cancer Biomarkers.” ACS Nano 15, no. 11: 18192–18205. 34735133 10.1021/acsnano.1c07075 PMC9286313

[jev270201-bib-0048] Piffoux, M. , A. K. A. Silva , C. Wilhelm , F. Gazeau , and D. Tareste . 2018. “Modification of Extracellular Vesicles by Fusion With Liposomes for the Design of Personalized Biogenic Drug Delivery Systems.” ACS Nano 12, no. 7: 6830–6842. 29975503 10.1021/acsnano.8b02053

[jev270201-bib-0049] Ramanathan, S. , B. B. Shenoda , Z. Lin , et al. 2019. “Inflammation Potentiates miR‐939 Expression and Packaging Into Small Extracellular Vesicles.” Journal of Extracellular Vesicles 8, no. 1: 1650595. 31489147 10.1080/20013078.2019.1650595 PMC6713176

[jev270201-bib-0050] Rankin‐Turner, S. , P. Vader , L. O'Driscoll , B. Giebel , L. M. Heaney , and O. G. Davies . 2021. “A Call for the Standardised Reporting of Factors Affecting the Exogenous Loading of Extracellular Vesicles With Therapeutic Cargos.” Advanced Drug Delivery Reviews 173: 479–491. 33862168 10.1016/j.addr.2021.04.012 PMC8191593

[jev270201-bib-0051] Ratajczak, J. , K. Miekus , M. Kucia , et al. 2006. “Embryonic Stem Cell‐Derived Microvesicles Reprogram Hematopoietic Progenitors: Evidence for Horizontal Transfer of mRNA and Protein Delivery.” Leukemia 20, no. 5: 847–856. 16453000 10.1038/sj.leu.2404132

[jev270201-bib-0052] Rayamajhi, S. , T. D. T. Nguyen , R. Marasini , and S. Aryal . 2019. “Macrophage‐Derived Exosome‐Mimetic Hybrid Vesicles for Tumor Targeted Drug Delivery.” Acta Biomaterialia 94: 482–494. 31129363 10.1016/j.actbio.2019.05.054

[jev270201-bib-0053] Royo, F. , U. Cossío , A. Ruiz de Angulo , J. Llop , and J. M. Falcon‐Perez . 2019. “Modification of the Glycosylation of Extracellular Vesicles Alters Their Biodistribution in Mice.” Nanoscale 11, no. 4: 1531–1537. 30623961 10.1039/c8nr03900c

[jev270201-bib-0054] Saleh, A. F. , E. Lázaro‐Ibáñez , M. A.‐M. Forsgard , et al. 2019. “Extracellular Vesicles Induce Minimal Hepatotoxicity and Immunogenicity.” Nanoscale 11, no. 14: 6990–7001. 30916672 10.1039/c8nr08720b

[jev270201-bib-0055] Stam, J. , S. Bartel , R. Bischoff , and J. C. Wolters . 2021. “Isolation of Extracellular Vesicles With Combined Enrichment Methods.” Journal of Chromatography B: Analytical Technologies in the Biomedical and Life Sciences 1169: 122604. 33713953 10.1016/j.jchromb.2021.122604

[jev270201-bib-0056] Sun, L. , M. Fan , D. Huang , et al. 2021. “Clodronate‐Loaded Liposomal and Fibroblast‐Derived Exosomal Hybrid System for Enhanced Drug Delivery to Pulmonary Fibrosis.” Biomaterials 271: 120761. 33774524 10.1016/j.biomaterials.2021.120761

[jev270201-bib-0057] Sutaria, D. S. , M. Badawi , M. A. Phelps , and T. D. Schmittgen . 2017. “Achieving the Promise of Therapeutic Extracellular Vesicles: The Devil Is in Details of Therapeutic Loading.” Pharmaceutical Research 34, no. 5: 1053–1066. 28315083 10.1007/s11095-017-2123-5 PMC5565485

[jev270201-bib-0058] Toh, W. S. , R. C. Lai , B. Zhang , and S. K. Lim . 2018. “MSC Exosome Works Through a Protein‐Based Mechanism of Action.” Biochemical Society Transactions 46, no. 4: 843–853. 29986939 10.1042/BST20180079 PMC6103455

[jev270201-bib-0059] Tsai, S. J. , N. A. Atai , M. Cacciottolo , et al. 2021. “Exosome‐Mediated mRNA Delivery In Vivo Is Safe and Can be Used to Induce SARS‐CoV‐2 Immunity.” Journal of Biological Chemistry 297, no. 5: 101266. 34600888 10.1016/j.jbc.2021.101266 PMC8483990

[jev270201-bib-0060] Usman, W. M. , T. C. Pham , Y. Y. Kwok , et al. 2018. “Efficient RNA Drug Delivery Using Red Blood Cell Extracellular Vesicles.” Nature Communications 9, no. 1: 2359. 10.1038/s41467-018-04791-8 PMC6004015 29907766

[jev270201-bib-0061] Vaidya, A. , S. Moore , S. Chatterjee , et al. 2024. “Expanding RNAi to Kidneys, Lungs, and Spleen via Selective ORgan Targeting (SORT) siRNA Lipid Nanoparticles.” Advanced Materials 36, no. 35: e2313791. 38973655 10.1002/adma.202313791 PMC11823468

[jev270201-bib-0062] Wang, Q. , J. Yu , T. Kadungure , J. Beyene , H. Zhang , and Q. Lu . 2018. “ARMMs as a Versatile Platform for Intracellular Delivery of Macromolecules.” Nature Communications 9, no. 1: 960. 10.1038/s41467-018-03390-x PMC5840177 29511190

[jev270201-bib-0063] Wang, X. , O. Omar , F. Vazirisani , P. Thomsen , and K. Ekström . 2018. “Mesenchymal Stem Cell‐Derived Exosomes Have Altered MicroRNA Profiles and Induce Osteogenic Differentiation Depending on the Stage of Differentiation.” PLoS ONE 13, no. 2: e0193059. 29447276 10.1371/journal.pone.0193059 PMC5814093

[jev270201-bib-0064] Wang, X. , F. A. Shah , F. Vazirisani , et al. 2020. “Exosomes Influence the Behavior of Human Mesenchymal Stem Cells on Titanium Surfaces.” Biomaterials 230: 119571. 31753474 10.1016/j.biomaterials.2019.119571

[jev270201-bib-0065] Wang, X. , and P. Thomsen . 2021. “Mesenchymal Stem Cell‐Derived Small Extracellular Vesicles and Bone Regeneration.” Basic & Clinical Pharmacology & Toxicology 128, no. 1: 18–36. 32780530 10.1111/bcpt.13478 PMC7820981

[jev270201-bib-0066] Williams, A. , H. Branscome , F. Kashanchi , and E. V. Batrakova . 2025. “Targeting of Extracellular Vesicle‐Based Therapeutics to the Brain.” Cells 14, no. 7: 548. 40214500 10.3390/cells14070548 PMC11989082

[jev270201-bib-0067] Yáñez‐Mó, M. , P. R.‐M. Siljander , Z. Andreu , et al. 2015. “Biological Properties of Extracellular Vesicles and Their Physiological Functions.” Journal of Extracellular Vesicles 4: 27066. 25979354 10.3402/jev.v4.27066 PMC4433489

[jev270201-bib-0068] Zhou, Q. , Y. Yan , Y. Li , et al. 2022. “Tumor‐Derived Extracellular Vesicles in Melanoma Immune Response and Immunotherapy.” Biomedicine & Pharmacotherapy 156: 113790. 36244269 10.1016/j.biopha.2022.113790

[jev270201-bib-0069] Zhou, X. , Y. Miao , Y. Wang , et al. 2022. “Tumour‐Derived Extracellular Vesicle Membrane Hybrid Lipid Nanovesicles Enhance siRNA Delivery by Tumour‐Homing and Intracellular Freeway Transportation.” Journal of Extracellular Vesicles 11, no. 3: e12198. 35233952 10.1002/jev2.12198 PMC8888792

[jev270201-bib-0070] Zhuo, Y. , Z. Luo , Z. Zhu , et al. 2024. “Direct Cytosolic Delivery of siRNA Via Cell Membrane Fusion Using Cholesterol‐Enriched Exosomes.” Nature Nanotechnology 19, no. 12: 1858–1868. 10.1038/s41565-024-01785-0 39300226

[jev270201-bib-0071] Zickler, A. M. , X. Liang , D. Gupta , et al. 2024. “Novel Endogenous Engineering Platform for Robust Loading and Delivery of Functional mRNA by Extracellular Vesicles.” Advanced Science (Weinheim) 11, no. 42: e2407619. 10.1002/advs.202407619. PMC11558116 39246205

