# Supplementary material for: Hybrid Extracellular Vesicles for Efficient Loading and Functional Delivery of mRNA
Source: J Extracell Vesicles. 2025 Dec 14;14(12):e70201. doi: 10.1002/jev2.70201 (PMC12703132; doi:10.1002/jev2.70201)
Supplement: Supplementary file 1 — Supplementary Material: jev270201‐sup‐0001‐SuppMat.docx [file JEV2-14-e70201-s001.docx]

**Supplementary Figures**

**
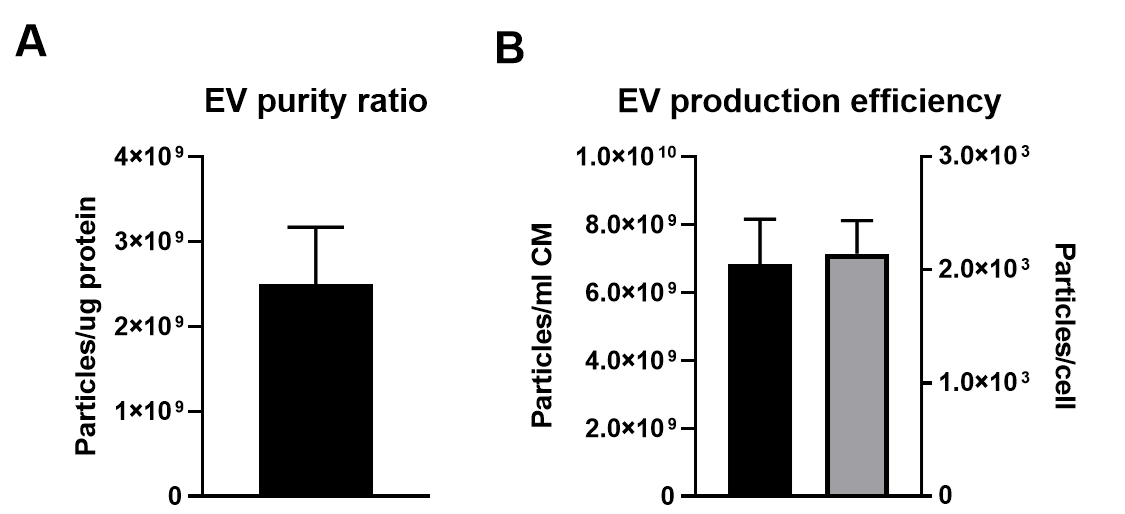
**

**Suppl. Fig.1 Expi293F cell derived EV preparation by UCF.**

(A) EV purity ratio indicated by particles per ug protein. (B) EV production efficiency indicated by particles per ml CM and particles per cell, respectively. Data is presented as mean ± SEM (n = 4).


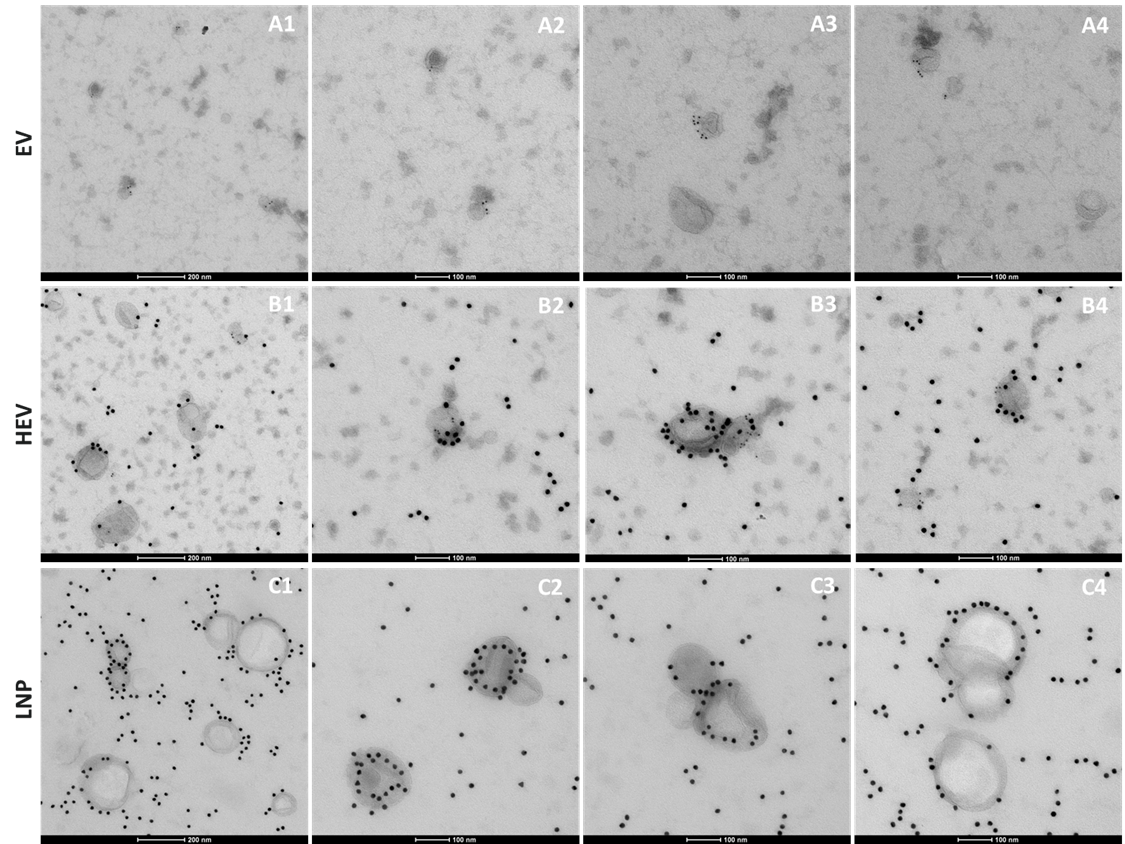


**Suppl. Fig.2 Immunoelectron microscopy images of EV, HEV and LNP dual-labelled with anti-CD63 and anti-PEG.**

(A1-A4) Images of EV, (B1-B4) images of HEV and (C1-C4) images of LNP at two different magnifications, A1, B1 and C1 at lower magnification and A2-A4, B2-B4 and C2-C4 at slightly higher magnification. Detection of CD63 and PEG are indicated by presence of 6 nm and 15 nm gold particles, respectively.

**Suppl. Fig.3 Mean size of EV and HEV measured by NanoFCM.**


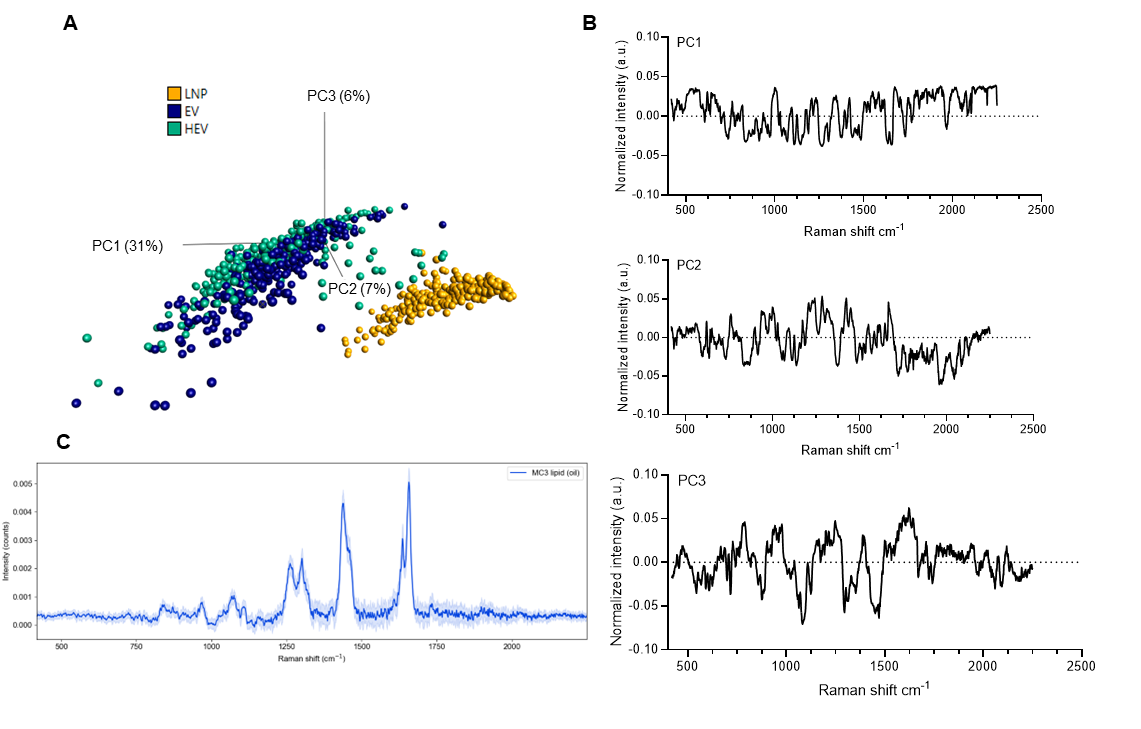


**Suppl. Fig.4 Multivariant analysis of Raman spectrum of EV, LNP and HEV.**

(A) Principal component analysis (PCA) clustering of Raman spectrum of EV, LNP and HEV. (B) Top three principal components. (C) Reference Raman spectrum of MC3 ionizable lipid in oil.

**Suppl. Fig.5 Quantification of RNAs associated with EVs and contribution of EV-associate RNAs in HEV productions .**

(A) Total RNA concentration and encapsulation efficiency of EV-associated RNAs. (B) Contribution of EV-associate RNAs in HEV productions. Data is presented as mean ± SEM (n = 3).


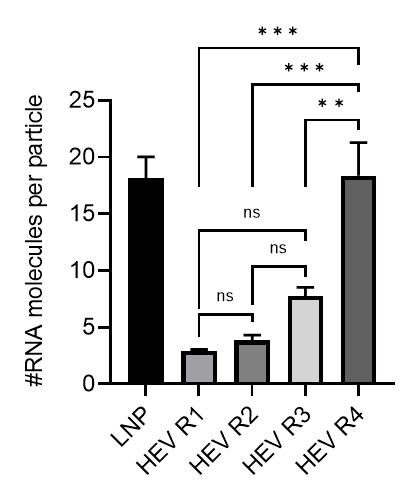


**Suppl. Fig.6 Estimation of RNA molecules in different HEV formulations.**

RNA amount in each group of nanoparticles was normalized to particle number and converted to molecule number by theoretical calculation based on the molecular weight of Cre mRNA. All data is presented as mean ± SEM, n = 3 biological replicates. * indicates statistically significant, where *** p-value < 0.001, ** p-value < 0.01, ns, non-significant.


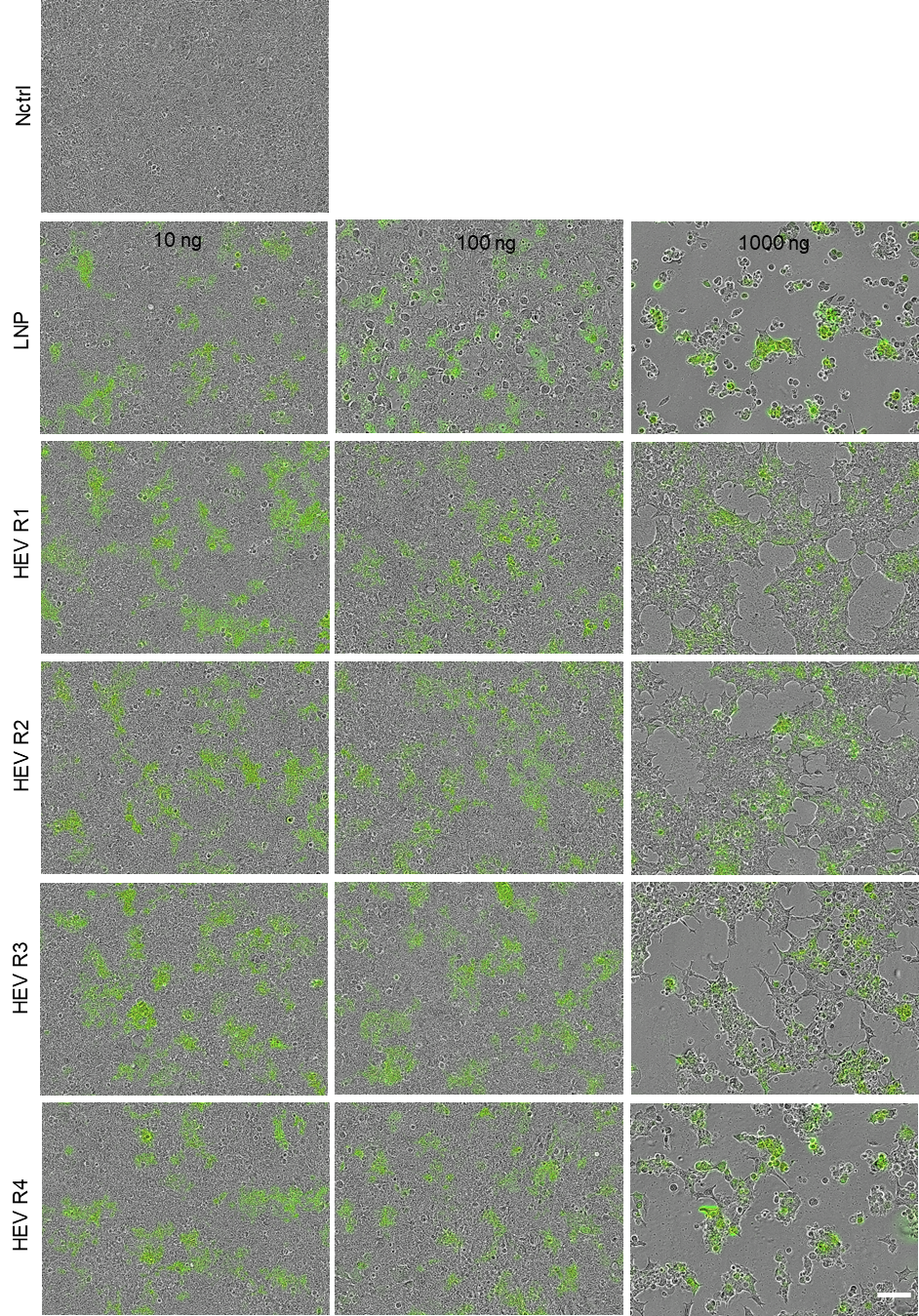


**Suppl. Fig.7 HEVs functionally deliver Cre mRNA to switch on EGFP expression in Cre reporter cells.**

Representative microscopic images show EGFP expression of cells upon HEV treatment at dose of 10, 100 and 1000 ng RNA per well for 72 h, respectively. Cells treated with PBS or LNP served as controls in the experiment. Scale bar, 100 µm.


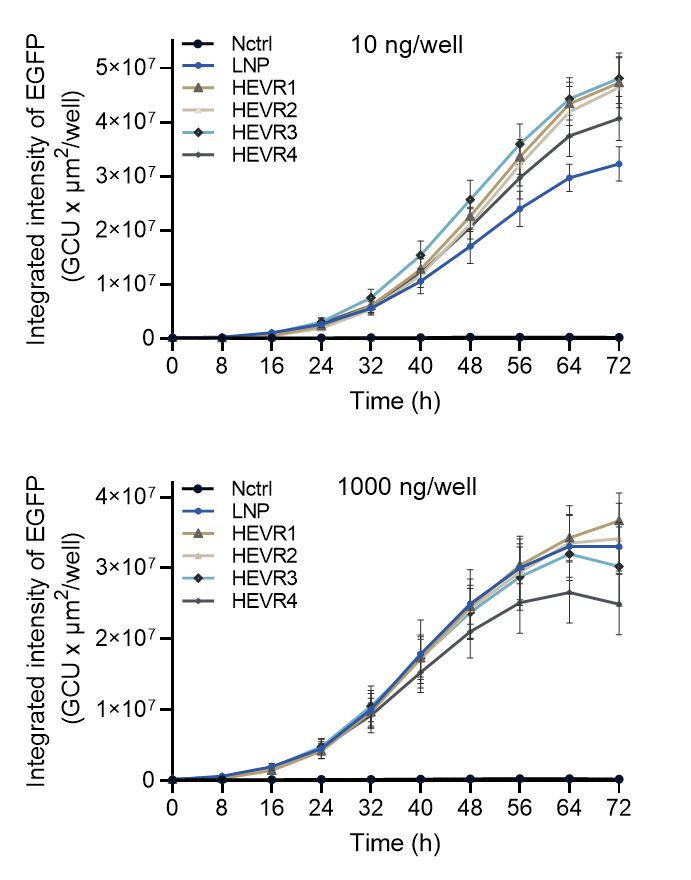


**Suppl. Fig.8 Quantification of EGFP expression in Cre reporter cells upon HEV delivery over 72h.**

Kinetics of EGFP expression upon cell treatment with HEVs at dose of 10 and 1000 ng RNA per well. Results are presented as total integrated fluorescence intensity of EGFP per well. Treatment with PBS or LNP served as controls in all experiments.
